# Improving prediction region accuracy in marine animal movement with temporal fusion transformer

**DOI:** 10.1038/s41598-025-29520-2

**Published:** 2025-12-04

**Authors:** Jorge Medina Hernández, Jorge P. Rodríguez, Clive R. McMahon, Ana M. M. Sequeira, Víctor M. Eguíluz

**Affiliations:** 1 Instituto de Física Interdisciplinar y Sistemas Complejos IFISC (CSIC-UIB), 07122 Palma de Mallorca, Spain; 2 CA UNED Illes Balears, Universidad Nacional de Educación a Distancia (UNED), 07009 Palma de Mallorca, Spain; 3 Sydney Institute of Marine Science, 19 Chowder Bay Road, Sydney, NSW 2088 Australia; 4 Department of Biological Sciences, Macquarie University, Sydney, NSW 2109 Australia; 5 Institute for Marine and Antarctic Studies, University of Tasmania, Private Bag 129, Hobart, TAS 7001 Australia; 6 Research School of Biology, Australian National University, Canberra, ACT 2600 Australia; 7 School of Biological Sciences and The UWA Oceans Institute, University of Western Australia, Perth, WA 6009 Australia

**Keywords:** Marine animal movement, Spatiotemporal forecasting, Machine learning, Satellite telemetry, Probabilistic forecasting, Animal migration, Computational science

## Abstract

Predicting marine animal movements from satellite tracking data remains challenging, limiting conservation and ecosystem management efforts. To address this, we trained the Temporal Fusion Transformer (TFT) neural network on tracking data from 434 southern elephant seals to forecast locations and fill data gaps (imputation) within 7-day windows. Compared to state-space models, TFT reduced location errors by 15% and produced more efficient prediction regions, identifying where seals were likely to be found while using less area: a fivefold reduction for forecasting and 30–40% reduction for imputation. The model performed best near the continental shelf and at low-to-moderate movement speeds, with bathymetry, water temperature and current direction being the most influential environmental factors affecting the model output. When applied to new geographic regions not represented in the training dataset, model performance declined by approximately 30% across most evaluation metrics, indicating challenges in transferring learned patterns to unfamiliar environments. Our findings show that deep learning is a promising tool for analyzing large, sparse tracking datasets. The enhanced predictive capabilities have potential for dynamic conservation measures, such as forecasting the spatial evolution of animals to minimize conflicts with human activities and environmental disturbances.

## Introduction

Marine animal movement provides information on the locations of marine species over time. The movement patterns of these animals are driven by a wide variety of factors, including environmental variables^1^, the spatiotemporal distribution of food^2,3^, avoidance of predators^4^ and breeding^5^. Consequently, movement constitutes a vital process supporting key ecological and biological functions. Tracking movement patterns can help identify areas where habitats overlap with threats. In the North Atlantic, for instance, up to 80% of shark habitats intersect with longline fishing zones^6^, increasing their exploitation risk. Globally, this overlap averages 24% but can reach 76% in certain regions^7^. Similarly, ship strikes are a major threat to marine mammals, but understanding their movement patterns–for example, identifying where migratory corridors are^8^–can help mitigate this risk. Tracking data from West Indian manatees revealed extensive activity in the northern Gulf of Mexico^9^, a region previously considered minor habitat. This information led to the re-design of boating corridors, reducing ship strikes. However, movement patterns can shift over time due to factors such as seasonality, habitat degradation^10^, and climate change^11^, highlighting the need for predictive models. Tools like TurtleWatch, which provides near real-time data on sea turtle habitats, have effectively reduced bycatch and supported dynamic fisheries management^12^. Thus, accurately tracking and predicting marine animal movements is essential for shaping conservation policies that protect species and their habitats^13–15^.

Traditional approaches to studying marine animal distribution and movement include species distribution models (SDMs) and state-space models (SSMs), respectively. SDMs predict species’ geographic distributions by combining occurrence data with environmental factors such as sea surface temperature and salinity, and are especially useful for assessing habitat shifts due to climate change. For instance, SDMs effectively modeled the distribution of the highly mobile western Arctic bowhead whale^16^; and estimated the impacts of climate change and fishing on Baltic cod, guiding sustainable exploitation strategies^17^. However, certain insights require analyzing the animal trajectories at the individual level, such as the evaluation of route fidelity^18^, the modeling of 3D movements^19^ or the assessment of behavioral states^20,21^. SSMs are able to model single animal trajectories, distinguishing between the location uncertainty and the actual movement process^22^. These models can incorporate both static and time-varying parameters^23^, capturing changes in behavior (e.g., resting, foraging, or traveling) in response to environmental factors or human disturbances, such as motorboat noise^24^. SSMs also enable real-time forecasting of movements^25,26^, which is crucial for managing human-animal interactions and prevent vessel collisions with endangered species. A subset of SSMs, Hidden Markov models (HMMs), are widely used in marine ecology. They rely on categorical state variables rather than continuous ones and follow the Markov property, meaning the future state depends only on the present, not past events. HMMs have shown high accuracy in classifying animal behavior states from simulated tracking data when there are clear differences in step lengths and turning angles between states^27^.

In recent years, machine learning models have gained prominence in the study of animal movements^28^. Using tri-axial accelerometry data, methods like support vector machines and decision trees have shown superior performance over linear models in classifying animal behavioral modes^29^. Additionally, acoustic telemetry data has been effectively applied to uncover spatiotemporal predator–prey overlap patterns^30^ and identify potential reproductive habitats^31^. Deep learning models also surpass traditional parametric models for one-step predictions, while stochastic differential equations remain more effective for long-range simulations ^32^.

Building on the success of deep neural networks in handling sequential data^33–35^, our aim is to evaluate their performance in forecasting marine animal movements and imputing missing tracking data. Similar to probabilistic weather forecasting, which must account for uncertainties in atmospheric conditions to predict extreme events and trends^36^, forecasting marine animal movements requires models that capture uncertainties stemming from dynamic animal behavior and environmental factors. Hence, beyond predicting location coordinates, we compute prediction regions (PRs) to estimate the likely range of movement, providing both a confidence measure for point predictions and insights into the extent of plausible movement at specific time points. We employ the Temporal Fusion Transformer (TFT)^37^, a transformer-based model ^38^ successfully used in fields like power grid management^39^ and healthcare^40^. The model uses a multi-horizon forecasting approach, optimizing predictions for specific discrete times within a limited time window. Thus, the extent of the model’s predictions is limited to a specific time horizon, at the exchange of being optimized specifically for being accurate at the target time steps. The model outputs quantiles for the unknown univariate distributions of Mercator coordinates (*X*, *Y*) at the target time step $t_{i}$. These coordinates are recorded through an ARGOS tagging system, and hence have an error determined by their class. The median serves as the point prediction, while the other quantiles define prediction regions (PRs) by stacking equally-tailed prediction intervals for each coordinate. However, this method is not optimized for minimizing the PR area, as it does not account for coordinate dependence and asymmetries in the univariate distribution tails. To address this, we estimate the bivariate probability density, referred to as TFT[B] in the results, and compute the highest density region (HDR^41^) that encompasses the target probability occupying minimal area. The process involves reconstructing univariate distributions from quantiles using quantile-respectful density estimation (QRDE^42^) and modeling their dependence with a Gaussian copula, where the correlation parameter is predicted by a separate TFT model. To adapt the model to our tasks, we modified the attention mechanism to account for the intermittent nature of tracking data by relying only on observed locations. Additionally, for the imputation task, we introduced layers that allow processing the input data symmetrically in both time directions.

Southern elephant seals, whose movement combines a phase of complex search with another of long-distance memory-driven migration^43^, are ideal species to test our methodology. Thus, we compare TFT to traditional state-space models (SSMs)^44^ such as Random Walk (RW), Move-Persistence (MP^45^), and Correlated Random Walk (CRW); at predicting unknown locations of southern elephant seals within time windows of 7 days, with a step of 6 hours. As a baseline, we include a Naive model that predicts the last observed location for the forecasting task and assumes a straight-line trajectory with constant speed between gap endpoints for the imputation task. The Naive model estimates prediction regions (PRs) based on the spread of past displacements. Our evaluation focuses on the accuracy of point predictions and the quality of the PR, which should ideally contain the true location while minimizing the area of uncertainty.

## Results

### Experimental design

**Fig. 1:**
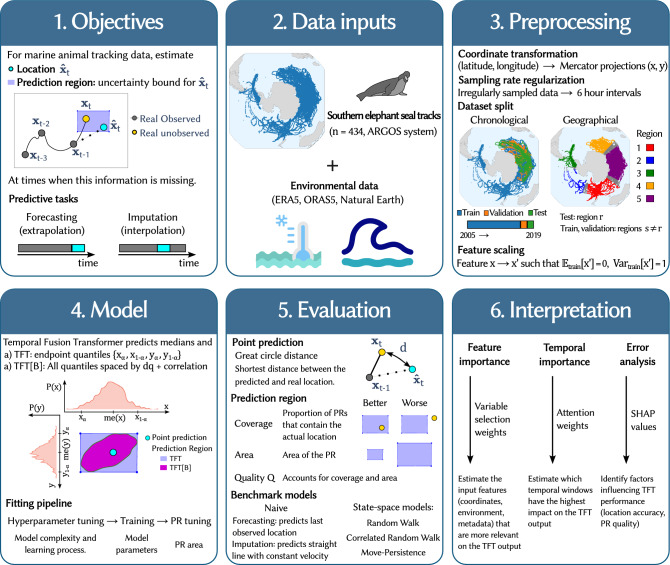
Methodology scheme for marine animal location prediction using temporal fusion transformer (TFT). (1) Objectives: define predictive tasks for estimating missing locations through interpolation and forecasting; (2) Data inputs include southern elephant seal tracking data (n=434, ARGOS system) and environmental variables (ERA5, ORAS5, Natural Earth); (3) Preprocessing involves coordinate transformation to Mercator projection, regularization to 6-hour intervals, feature scaling, and dataset splitting using geographical (spatial cross-validation across regions 1-5) and chronological approaches (2005–2019); (4) Model implementation uses TFT architecture with hyperparameter tuning and prediction region (PR) calibration, producing both point predictions and uncertainty bounds through endpoint quantiles (TFT) or full quantile distributions with correlation (TFT[B]). In the prediction region visualization, TFT[B] is presented to produce lower area. This is true in theory, although in practice it has to predict more quantiles, which lead to more error accumulation and potentially lower performance; (5) Evaluation employs the great-circle distance to measure the location error and prediction region quality to assess the proportion of PRs containing actual locations while accounting for prediction area. These results are benchmarked against state-space models (Random Walk, Correlated Random Walk, Move-Persistence) and a Naive model; (6) Interpretation analyses include determining which features and temporal windows contribute more to the model output, as well as the factors that influence model performance. The latter is performed by computing the SHAP values of an explanation model (gradient-boosted trees) predicting the performance metrics of TFT.

This study evaluates the effectiveness of Temporal Fusion Transformer (TFT) for predicting the movement of southern elephant seals. The process involves several key stages, as outlined in the methodology scheme (Fig. 1).

**Fig. 2:**
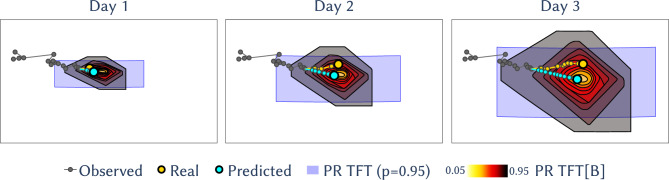
Forecasting task overview. TFT and TFT[B] use historical location data and environmental features from past time steps $i = -M,\dots , 0$ as input (gray dots) to predict location quantiles for future time steps $i = 1,\dots , n$. Actual future locations $\mathbf {x_{i}}$ are depicted in yellow. For each future step, the models generate a predicted location $\hat{\textbf{x}_{i}}$ (cyan) along with additional quantiles that define prediction regions (PRs) targeting a coverage error rate $\alpha _{F}$. PRs are represented as shaded areas: TFT’s PR with target coverage probability $p=1-\alpha _{F}=0.95$ is plotted in blue, while PRs from TFT[B] –derived from the highest-density regions of the bivariate probability density– range from yellow ($p = 0.05$) to black ($p = 0.95$) in 0.1 increments. The markers for the predicted and actual locations are enlarged for the final time step in each subfigure. The objectives are to (1) minimize the distance between $\hat{\textbf{x}_{i}}$ and $\mathbf {x_{i}}$, and (2) adjust the PR shape to achieve the desired CER $\alpha _{F}$ with minimal area. Additional example trajectories are shown in Fig. S11.

**Fig. 3:**
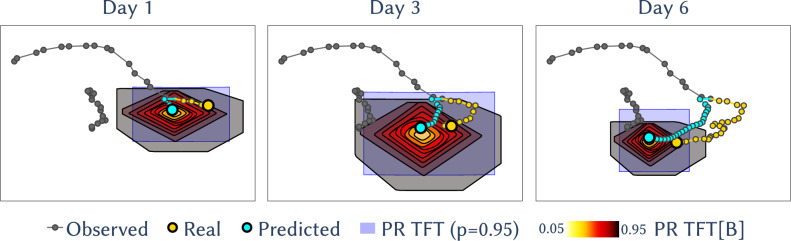
Imputation task overview. TFT and TFT[B] take as input location data and environmental features from past ($i = -M,\dots ,0$) and future ($i = n+1,\dots , N$) time steps, and predict location quantiles for time steps within the imputation window ($i = 1,\dots ,n$). Actual locations $\mathbf {x_{i}}$ within the imputation window are depicted in yellow. For each time step, the models output a predicted location $\hat{\textbf{x}_{i}}$ (cyan) along with additional quantiles that define prediction regions (PRs) targeting a coverage error rate $\alpha _{F}$. PRs are represented as shaded areas: TFT’s PR with target coverage probability $p=1-\alpha _{F}=0.95$ is plotted in blue, while PRs from TFT[B] – derived from the highest-density regions of the bivariate probability density – range from yellow ($p = 0.05$) to black ($p = 0.95$) in 0.1 increments. The markers for the predicted and actual locations are enlarged for the final time step in each subfigure. The objectives are to (1) minimize the distance between $\hat{\textbf{x}_{i}}$ and $\mathbf {x_{i}}$, and (2) adjust the PR shape to achieve the desired CER with minimal area. Additional examples are shown in Fig. S12.

The primary goals were to obtain accurate location predictions and uncertainty bounds (prediction regions) for these locations, for two specific tasks: forecasting future positions (Fig. 2) and imputation of missing data points within a trajectory (Fig. 3). The former constitutes an extrapolation in the time domain, while the latter is an interpolation.

The model was trained on a dataset comprising 434 southern elephant seal trajectories recorded via the ARGOS satellite system. The input data for the model consisted on the tracking data, containing the spatiotemporal trajectories and their associated metadata, complemented by external environmental data. To prepare the data for the TFT model, a series of preprocessing steps were performed. All tracking data was binned into discrete 6-hour time intervals. Latitude and longitude coordinates were converted to their Mercator projections to simplify modeling. Finally, features were scaled, and highly correlated environmental variables were filtered to improve model stability.

We considered two dataset splits to analyze two different aspects of the model performance: the validity of past movement patterns to predict future ones (chronological split) and its generalization capabilities to unseen geographical regions (geographical split). In the chronological split the model is trained on the oldest trajectories, and tested in the newest ones, while in the geographical split the model is trained across several spatial areas and tested in an unseen region.

The TFT model outputs quantiles of the distributions of locations for the specified time. We use the median of these distributions as an estimate for the location (point prediction), and train two different model variants differing in the way the prediction regions are computed. In the standard version (TFT), the model is trained on the endpoint quantiles for each dimension associated to the desired confidence level, which results on rectangular PRs. In contrast, the TFT[B] variant predicts all quantiles of the location distributions with a specified resolution, from which we infer the joint bivariate distribution of coordinates (see Methods). Then, the PRs correspond to the area of the 2D distribution encompassing the target probability with minimal area.

To evaluate the accuracy of point predictions, we compute the great circle distance between the actual and predicted locations. For assessing prediction regions, we use the proposed quality score *Q*, which balances two objectives: containing the real location with the desired coverage probability, and doing so while employing minimal area. We benchmark the performance of TFT and TFT[B] against traditional state-space models (SSMs), such as Random Walk (RW), Correlated Random Walk (CRW) and Move-Persistence (MP). We also include a Naive model, which predicts the last observed location in the forecasting task and a straight line with constant velocity in the imputation task. For every model, we tune the area of the predicted regions using the validation data (see Methods), which lead to better quality scores.

To interpret the trained TFT predictions, we analyzed its variable selection weights to identify the most influential environmental features and examined attention weights to determine the most relevant time windows. We then trained an explanation model (gradient boosted trees) to predict the distance error and prediction region quality of the TFT outputs, and used its SHAP values to pinpoint the factors with the greatest impact on model performance.

### Chronological split

#### Forecasting

TFT and TFT[B] significantly outperform all models in terms of average point error, with only the difference with MP (a 17% decrease) being not statistically significant (Table 1). However, when trained on single trajectories (TFT[s]) the distance error is the highest, comparable only to CRW. Most models struggle to beat Naive, with only TFT, TFT[B] and MP doing so with statistical significance. Across time steps, TFT and TFT[B] consistently show the lowest distance error (Fig. S1a), though mostly not significantly different from MP and RW (Table S1); and are the only models outperforming Naive with statistical significance. For prediction regions, TFT and TFT[B] match other models in coverage error rate (CER) but use significantly less area (Fig. S2a), leading to a higher quality score *Q* (Table 1). Specifically, TFT requires about 2.5 times less area for PRs with target coverage error rates of $\alpha _F = 0.05$ and $\alpha _F = 0.1$ compared to the best performing SSM (MP), and 60% more area for $\alpha _F = 0.5$ (Table S2). For TFT[B], the improvements are higher, with a reduction of around 5 times less area for $\alpha _{F}=0.05$ and $\alpha _{F}=0.1$, while having a similar area for $\alpha _{F}=0.5$. As with distance error, TFT and TFT[B] are the only model to significantly outperform Naive, while TFT[s] has among the lowest quality scores and the lowest CER quality $Q_{\alpha }$. Notably, TFT achieves a performance comparable to the best SSM for $n_{\textrm{train}}=25$ and $n_{\textrm{train}}=10$ training trajectories in terms of distance error and PR quality, respectively; and surpasses it in PR quality for training sizes of at least $n_{\textrm{train}}=100$ trajectories, but not in distance error with statistical significance for $n_{\textrm{train}}<356$ (Fig. S3a).

According to variable selection weights, the most important features for predicting future locations are the past location coordinates (Fig. S4a). Other key contributors are time features (day, month, season), the sampling period or time between observations, and environmental factors such as bathymetry, temperature and salinity. The most relevant static variable is the animal’s sex. The attention scores give more weight to time steps further in the past, with the first input day contributing about half of the cumulative attention (Fig. S5a).

**Fig. 4:**
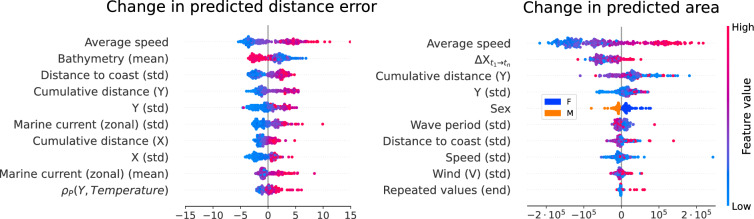
Predictors of TFT Performance for the Forecasting Task. SHAP values for gradient boosted trees regression of the point prediction error in km (left) and prediction region area in km$^{2}$(right). Input includes trajectory characteristics and average, standard deviations and correlations among the TFT input features. $\rho _{P}$ denotes the Pearson correlation. Features are ordered by average absolute SHAP value, with a color spectrum from blue (lower values) to red (higher values) for numerical features, and solid colors for categorical features. Distance regression achieves a 9% RMSE reduction relative to the baseline in the test set and an 18% in the validation set, while area regression achieves 19% and 72% in the test and validation sets. Point prediction and PR area are interconnected, with a Spearman correlation of 0.57 (Fig. S7a), reflected in variables having similar influences on model output. Optimal performance occurs along the continental shelf (depth < 1000 m), and when the animal had not moved at high speeds prior to the prediction window.

Based on the SHAP values for the explanation model, the most influential factors for predicting TFT’s point accuracy are the animal’s average speed, bathymetry, and variables that quantify the traveled distance (Fig. 4). Specifically, the explanation model predicts optimal performance when the animal is not moving at high speeds and within the continental shelf (depth < 1000 m), which is in agreement with the test set statistics (Fig. S6a). For trajectories with average speed below 3 km/h (80% of the data) and depth smaller than 1,000 m, the average distance error is 32 km (C.I. [24, 44] km), compared to 91 km (C.I. [81, 103] km) for other trajectories (Fig. S6b). The correlation analysis points to similar conclusions (Fig. S7a), and indicates the performance of the point prediction and the PR are interconnected, with a Spearman correlation between the distance error and the quality score of − 0.495. Thus, some of the most relevant predictions for the PR area coincide with those for the distance error. A natural question is whether the drop in performance for deep areas and high speeds occurs due to a genuine increase in the randomness of the movement in those circumstances, or a lack of training data to learn from. Partially, it can be explained by the data availability, as evidenced by a Spearman correlation of -0.56 between distance error in the test set and the kernel density estimate for training data (Fig. S6a).

#### Imputation

Regarding distance error, TFT and TFT[B] outperform all SSMs model with statistical significance (Table 2). Specifically, they achieve an error 14% lower than the best performing SSM (RW). Notably, none of the models outperform the straight-line prediction of the Naive model with statistical significance, with CRW(+55% error) and MP (+16% error) having a statistically significant worse performance, and TFT outperforming Naive by 12% without significance. Across time steps, TFT and TFT[B] provide the lowest distance error (Fig. S1b), although the difference is not statistically significant from not Naive and RW (Table S1). For prediction regions, TFT and TFT[B] have similar performances to SSMs in terms of CER while using less area (Fig. S2b), and hence achieve a higher quality score Q. Compared to the best performing SSM (RW), they use 36-43% less area for $\alpha _{F}=0.05$, 30–35% for $\alpha _{F}=0.1$ and a similar area for $\alpha _{F}=0.5$ (Table S3). As in the forecasting task, TFT trained on a single trajectory (TFT[s]) obtains the worst results in both distance error and PR quality. Furthermore, TFT requires more training trajectories than in forecasting to match and surpass SSMs. It equals the best SSM’s distance error at $n_{\text {train}}=50$ trajectories and its PR quality at $n_{\text {train}}=100$ trajectories; and outperforms it on both metrics at $n_{\text {train}}=356$ (Fig. S3b).

Similar to the forecasting task, the most relevant features for imputing locations are the spatial coordinates, followed by the temporal features and environmental factors, mainly bathymetry, temperature and marine current direction (Fig. S4b). However, the temporal distribution of attention differs, with time steps closer to the imputation window contributing more to the model output (Fig. S5b).

**Fig. 5:**
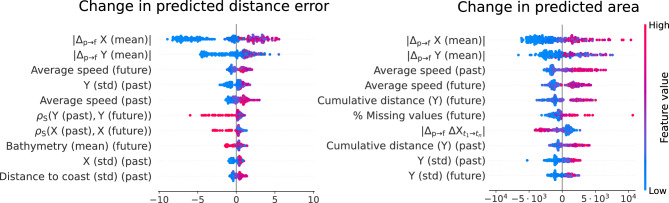
Predictors of TFT Performance for the Imputation Task. SHAP values for gradient boosted trees regression of point prediction error in km (left) and prediction region area in km$^{2}$(right). Input includes trajectory characteristics and average, standard deviations and correlations among the TFT input features. $\rho _{S}$ denotes the Spearman correlation, and $\Delta _{\textrm{p}\rightarrow \textrm{f}}$ indicates the difference in the feature when evaluated in the future compared to the past. Features are ordered by average absolute SHAP value, with a color spectrum from blue (lower values) to red (higher values) for numerical features, and solid colors for categorical features. Distance regression achieves an 18% RMSE reduction relative to the baseline in the test set and a 19% in the validation set, while area regression achieves 31% and 30% in the test and validation sets. Point prediction and PR area are interconnected, with a Spearman correlation of 0.77 (Fig. S7b), reflected in variables having similar influences on model output. As for the forecasting task, optimal performance is achieved along the continental shelf (depth < 1000 m) and for animals moving at low to intermediate speeds. However, for the imputation task, high movement similarity between both ends of the imputation window is the most relevant factor.

As highlighted by the SHAP values for the explanation model (Fig. 5), low to intermediate movement speed and movement located along the continental shelf are predictive of higher model accuracy, as in the forecasting task. However, the main predictors are the absolute differences in the coordinates between the input chunks before and after the imputation window, with lower differences predicting better performance. This is in agreement with the Spearman correlation analysis for the test set (Fig. S7b), which also implies an association between distance error and quality score (Spearman correlation = − 0.51), reflected in similar top predictive features for distance error and PR area.

### Geographical split

#### Forecasting

TFT and TFT[B] performed worse under geographical split compared to chronological split, where training and test data overlapped geographically, with distance error increasing 32% (not statistically significant) and prediction quality decreasing 31% (statistically significant).

Both TFT versions achieved an averaged distance error lower than Naive and CRW with statistical significance, lower RW without statistical significance, and similar to MP (Table 3). This trend is generally reflected across the individual realizations involving different test regions, although for Naive and CRW the differences lack statistical significance. For prediction region quality, both TFT variants scored highest with statistically significant improvements over other models.

#### Imputation

Similar to the forecasting task, prediction region quality decreased by 33% compared to the chronological split setting. However, the distance error worsened by a greater degree, increasing by 170% (Table 4). Both differences are statistically significant.

TFT and TFT[B] significantly underperformed all other models in distance error, primarily driven by test region #1, which showed 4-6 times greater error than other regions. In terms of prediction region quality, both TFT variants significantly outperformed all models except MP, which achieved similar scores.

## Discussion

The results show that TFT and its imputation adaptation outperform SSMs for both forecasting and imputation tasks when train and test data overlap geographically (chronological split). In this setting, TFT achieves at least a 17% reduction in distance error for forecasting (though not statistically significant compared to MP) and at least a 14% reduction for imputation, with statistical significance. The primary advantage of TFT lies in its ability to estimate prediction regions where an animal is likely to be with a given probability. TFT achieves the desired coverage error rate while employing less area compared to all SSMs: 30–40% less for 90–95% PRs in imputation, and a 2.5 fold reduction for the 90–95% PRs in forecasting. Furthermore, computing the bivariate distribution (TFT[B]) enhances forecasting performance, yielding an additional twofold reduction in the area of 90–95% PRs compared to standard TFT. However, these improvements are not maintained when TFT is evaluated in a test region not present in the training data (geographical split). Performance degradation approximates 30% across metrics and tasks, with imputation showing unstable behavior in terms of distance error, since one of the five test runs exhibited 4-6 times the average error. Despite this decline, TFT remained a competitive alternative for the forecasting task, delivering statistically significant improvements in prediction region quality while matching the best-performing model (MP) in distance error. On the contrary, in the imputation task TFT obtained worse distance error compared to all models with statistical significance, and a prediction region quality similar to the best performing model (MP), and superior to the rest with statistical significance.

According to the variable selection weights, the model relies mostly in the coordinate features of the input time steps. Among environmental factors, bathymetry, temperature and the direction of the marine currents had the greatest influence on the model output. These factors are well-documented as critical to the movement of various marine species^46–48^, including the southern elephant seals^49–51^, whose foraging routes correlate with temperature and bathymetric profiles^52^. Precisely the bathymetry, along with movement speed, plays an important role in determining TFT performance for new trajectories. The model performs best in terms of distance error and PR area along the continental shelf (depth < 1,000 m) and when animals move at low to intermediate speeds. The relation with speed is intuitive: predicting larger movement ranges is more challenging than predicting the movements of stationary animals, especially when speed and direction do not remain constant, with unpredictability compounding over time. On the other hand, the effect of the bathymetry on model performance might be linked to southern elephant seals restricting their movement to the continental shelf. For instance, juveniles on the Patagonian shelf tend to stay within shallow areas^53^, reducing the uncertainty in the movement. Additionally, regions of area-restricted search for southern elephant seals are highly concentrated on the Antarctic continental shelf^54^. One hypothesis to be explored is whether trajectories become less predictable during dives, given deeper areas are associated with longer and deeper dives^55^. Notably, females are associated to higher PR area, as pointed out by the explanation model (Fig. 4), which could be related to differences in behavior with respect to the bathymetry. For example, females from Kerguelen Island have been found to shift to deeper areas during winter, whereas males remain on the continental shelf^56^. For the imputation task, the most predictive feature was the absolute difference in coordinates before and after the imputation window, with smaller differences being linked to better performance. Moreover, these properties are predictive of both the distance error of point predictions and the PR area, which are themselves correlated (Spearman correlation = − 0.5). Thus, simultaneously predicting the PR along with the point location is a valuable tool to understand not only the plausible range of the animal movement but also the accuracy of the location estimate.

Beyond its predictive capabilities, a key advantage of TFT is its low inference time (less than a second per instance). This speed enables real-time forecasting, making it ideal for applications like analyzing data from elephant seal–borne sensors^57,58^ that track the state of the ocean. Integrating these insights can improve the planning of marine protected areas and other conservation efforts. Consequently, as these methods become available, the demand for accessible real-time data grows, which is crucial for deepening our understanding of ocean ecosystems and monitoring their health, including preserving diverse marine populations.

The reproducibility of this work, with all code publicly available on Github and all data accessible from established online repositories, facilitates the integration of TFT (or TFT[B]) as a modular component within larger computational frameworks. Thus, TFT would function as a dedicated module to probabilistic location forecasting, predicting the location and uncertainty areas where the animal may be, that can be placed at any point of the computation pipeline. It could be applied after a state-space model to ensure inputs are smoothed and regularly sampled, or serve as input to other software, such as a real-time decision system that adjusts shipping lanes to minimize vessel strikes. Additionally, the internal states of the model can serve as enriched feature inputs for downstream models. The system learns complex, high-dimensional representations (embeddings) that fuse an animal’s movement history with environmental context. These embeddings could be later processed by specialized models tailored to other goals, such as foraging success, stress levels, or breeding events.

Conversely, TFT has several limitations compared to SSMs. The most evident is its limited flexibility in prediction. Unlike SSMs, which model the movement of an animal as a latent continuous process, TFT is trained to predict within a fixed number of equally spaced time steps. This approach sacrifices the ability to make predictions at arbitrary time points, at the exchange of potentially more optimized predictions at specific intervals. Additionally, TFT does not account for tracking errors caused by device imprecision, unlike SSMs. Instead, the model predicts point locations and prediction regions for the average tracked location within a time bin, and hence the accuracy with respect to the real movement of the animal will ultimately depend on the resolution and regularity of the tracking itself. Geographic transferability presents another challenge. As analyzed in the geographical split setting, TFT struggles to transfer its performance to unseen regions, suggesting that some movement patterns contain region-specific characteristics. This geographical bias prevents the model from extracting truly generalizable features, a problem well-documented in tracking-based models. Species Distribution Models, which have different goals and methodologies but use similar input data, exhibit the same limitations: artificially inflated performance near tagging sites and reduced accuracy in novel areas^59,60^. Another limitation is the size of the data set required to outperform SSMs. While SSMs can be fitted to a single trajectory, TFT requires training on a dataset containing several hundred trajectories to enhance performance, at least in the context of the species and region studied. However, the scalability with dataset size may enable broader generalization as the model incorporates information from different sources, compared to the SSMs that are limited to single-trajectory fitting. Furthermore, transfer learning from other species and regions may help reduce the dataset size requirement.

Future work could address these limitations by implementing a neural differential equation (NDE^61^) model, where the trajectory evolution is parameterized by a neural network. This approach combines the strengths of both SSMs and neural networks. Like SSMs, NDEs operate in continuous time, enabling them to handle irregular time series without preprocessing steps (e.g., data binning) that may distort the data. Additionally, NDEs leverage the neural network capabilities for capturing non-linear dependencies and context-dependent dynamics, and efficiently aggregate knowledge across the training trajectories. Furthermore, the flexibility of the deep learning framework facilitates the addition of variables potentially relevant to the movement processes of marine animals; such as diving data (i.e., the depth coordinate) to capture the effect of vertical movement dynamics, the presence or absence of the same or related species (e.g., predators or prey), and human activities like boat traffic. To mitigate geographical bias, the model could be trained to learn region-invariant features using domain adversarial neural networks (DANN^62^). This technique couples a region discriminator with the feature generator through adversarial training: the discriminator attempts to identify geographical regions while the generator learns to deceive it by developing region-invariant features. The main challenges with this approach are defining what constitutes distinct regions and the extent to which region-invariant features can characterize animal movement. This study serves as an initial step toward more accurate probabilistic forecasting of animal movement, providing a foundation for further improvements.

## Methods

### Dataset

We use tracking data of southern elephant seals from French Polar Institute programs: SNO-MEMO^63^ (program 109: PI. H. Weimerskirch) and CNES-TOSCA^64^ (program 1201: P.I. C. Gilbert and C. Guinet); and from Australia’s Integrated Marine Observing System (IMOS^65^) (Table S4). The dataset contains 434 trajectories recorded between 2005 and 2019 using the ARGOS tagging system. The average duration is 182 days, and sex representation is balanced, with 204 trajectories being from females, 198 from males, and 32 from individuals of unknown sex. The tracking data is complemented with environmental features from ERA5^66^, ORAS5^67^ and Natural Earth^68^ (Tables S5–S9), known future information (Table S10) and metadata (Table S11). We selected environmental variables known to influence the movement of marine mammals, such as temperature, marine currents and wind speeds^69^, and added related variables (e.g. wave direction) that were available in the open datasets we used. Additionally, we included the bathymetry since there is compelling evidence that water depth consistently affects movement patterns across 50 marine vertebrate species^70^. Specifically, ballistic movement patterns were more prevalent in open ocean (depth $>150$m) compared to coastal areas (depth $<150$m), where search behavior was more frequent.

### Preprocessing

#### Coordinate transformation

To facilitate the comparison with aniMotum^44^ SSMs, we use the Mercator projection

1 $$\begin{aligned} {\left\{ \begin{array}{ll} x = \text {R}\phi \\ y = \text {R}\log \big (\tan (\frac{\pi }{4} + \frac{\tilde{\theta }}{2})\big ) \end{array}\right. }\quad \tilde{\theta } = \frac{\pi }{2} - \theta \end{aligned}$$

where $\phi \in [-\pi , \pi ]$, $\tilde{\theta }\in [-\pi /2, \pi /2]$ and $\theta \in [-\pi /2, \pi /2]$ are the longitude, latitude and polar angle, respectively, and R is the Earth radius in km.

We encode the continuous temporal information using the day of the year. Given it is a quasi-periodic variable, where large numeric differences can correspond to close points in reality, e.g. days 1 and 365, we decompose it into sine and cosine components

2 $$\begin{aligned} t \rightarrow (\sin \tilde{t}, \cos \tilde{t}), \quad \tilde{t} = \frac{2\pi }{T-1} (t-1) \end{aligned}$$

where *t* has units of days and its decimal part takes into account temporal variations up to the order of seconds, and *T* is the number of days in the year: 366 for leap years and 365 for normal years. Additionally, we use the hour angle to encode the local solar information. The solar hour angle represents the position of the sun in the sky at any given time of the day, at a given location. It corresponds to the angular displacement of the Sun east or west of the local meridian. It is negative (positive) before (after) the solar noon, when the Sun is directly above the meridian. We compute it using the ‘solarposition’ module of the ‘pvlib’ python package.

#### Feature selection

We filter geographical and environmental features by collinearity (Fig. S8). We compute the hierarchical clustering of Pearson correlations among environmental features, using the ’average’ method (UPGMA). Clusters include salinity (at 0 and 10m), temperature (at 0, 10, 97 and 1046m), zonal marine current (at 0, 97 and 1516m), meridional marine current (at 0, 97 and 1516m), solar radiation (solar radiation, UV radiation), and infrared albedo (diffuse and direct). We use one representative feature from each cluster.

#### Sampling rate regularization

The Temporal Fusion Transformer model does not handle irregularly-sampled time series. However, it does allow for missing values. Thus, trajectories are binned in time intervals with $\Delta t=6$ hours width, and values correspond to the mean of the feature in that interval. Missing time steps for numerical features are filled using the forward strategy, which replaces any missing value by the most recent previous value that is not missing. For categorical variables, missing values are grouped under a new category ‘missing’. Missing values occur both in the input and in the prediction window. Thus, the evaluation metrics and the attention scores are averaged over real observations only.

#### Dataset split

To evaluate the performance of TFT, we split the dataset into training, validation and test sets, each containing multiple trajectories. The training set is used by the model to optimize its parameters and learn the underlying data patterns. We use the validation set to prevent overfitting by halting training when validation error fails to improve in several consecutive iterations (*early stopping*). We also employ the validation set to optimize the model complexity (select the model hyperparameters) and tune the area of the predictions region after training. Finally, the test set provides an unbiased measure of the model performance, as its contents do not influence both parameter or hyperparameter fitting processes. Every other model (Naive, TFT[s], and SSMs) is trained individually for each trajectory in the test set, but uses the validation set for hyperparameter tuning, affecting the prediction regions. For SSMs, the forecasting task uses the last 7 days of each trajectory for evaluation, with the remainder for training. In the imputation task, 7-day missing windows are created, spaced 28 days apart, with the model trained on all remaining data and evaluated on the missing windows. The Naive model follows the same split as TFT, using 7 days for evaluation and the optimized number of input days (Table S12). For imputation, the same number of past and future days is used as input.

We perform two different strategies for splitting the dataset into training, validation and test sets, aiming to diminish the effect of two different types of biases: the look-ahead bias, where the model is trained on future information that would not be available at the time of prediction, and the geographical bias, where the patterns learned by the model do not generalize to other spatial regions.

To avoid look-ahead bias, we divide the data chronologically into training (80% oldest data), validation (10% data newer than training data), and test (10% newest data) sets (Fig. S9a). This split allows to assess whether past patterns can be useful in predicting future movements. For TFT trained on a single trajectory (TFT[s]) we include a validation region for assessing training performance, and allow overlapping among the input regions but not among the evaluation ones (Fig. S9b). This maximizes the available training data, while limiting data leakage. The remaining data that does not overlap with the validation evaluation is used for training.

However, the chronological split causes the training, validation and test set spatial distributions to overlap (Fig. S10a), and therefore does not allow to determine the generalization capabilities to new regions. Hence, we divide the data into five geographical regions (Fig. S10b), of equal area. The clusters are separated by buffer zones of width 10 degrees that are not assigned to any region. In this setting, one region is held out as a test set, and the remaining four regions are used as training and validation. We train and evaluate the models five times, using a different test region in each iteration, and report all the realizations and the averaged performance across test regions.

#### Feature scaling

We apply standard scaling to all environmental variables (Tables S5–S9), with scaling parameters fitted using the training set only. For the target variables, i.e. the Mercator projections (Eq. 1), we use encoder normalization. This method fits a standard scaler on the observed values of each trajectory, ensuring a consistent output range for the model and improved generalization across varying scales. Furthermore, since the fitting process occurs in past values, the encoder normalization avoids look-ahead bias for the forecasting task.

### Model

We use the Temporal Fusion Transformer (TFT^37^) with the quantile loss^71^ (Eq. 17) for estimating the quantiles of coordinates at a target time step (*x*(*t*), *y*(*t*)), independently for each dimension. The model is based on the transformer architecture and handles various types of input data: past information (encoder variables) such as geographical location (Table S4) and environmental variables (Tables S5–S9) and time related variables (Table S10), known information within the prediction window (decoder variables) (Table S10), and metadata (Table S11) that does not vary with time (static variables). Adept at time series forecasting, it uses the attention mechanism (Eq. 40) to capture temporal patterns and weight the input data. The model handles regularly sampled data only and generates output for a fixed number of future time steps (multi-horizon forecasting). We also present the results for TFT trained on single trajectories, referred to as TFT[s]. Prediction regions for TFT are calculated using equally-tailed prediction intervals for each coordinate. This approach, however, is not optimized to minimize the prediction region’s area. To address this, we compute the highest density region of the bivariate probability density, denoted as TFT[B] in the results section, where the univariate distributions are estimated via quantile respectful density estimation (QRDE^42^), and the dependence is modeled by a Gaussian copula. To adapt the model to the intermittent nature of tracking data, we modified the attention mechanism forcing it to rely only on observed locations, i.e. attention scores for missing values are masked to zero. For the imputation task, we introduced layers that process input data symmetrically in both time directions. In the forward pass, data flows in the natural order: encoder (past) $\rightarrow$ decoder (prediction window) $\rightarrow$ future, where each step uses its own LSTM layer and takes the context of the previous layer. This is concatenated along the feature dimension with the output of the backward pass: future $\rightarrow$ decoder $\rightarrow$ encoder. For consistency with the rest of the model, we set the output size of the variable selection networks, static-enriched initial context, and LSTM hidden cell to half the hidden size. Similarly, the input and output sizes of the LSTM layers are halved, resulting in a tensor of feature dimension ‘hidden size’ after the forward and backward outputs are concatenated.

We compare it to standard state-space models available at the aniMotum^44^ R package. These continuous-time models take as input the sequence of geographical locations and fit parameters for their evolution equations. Hence, they naturally handle irregularly sampled data, both in the input and output sides. Specifically, the SSMs considered are:

1. Random Walk (RW). It is a stochastic process where each step (distance and direction) is independent of the previous steps.
2. Correlated Random Walk (CRW). Each step is influenced by the direction of the previous step, introducing correlation between successive steps.
3. Move-Persistence (MP^45^). Estimates time-varying move persistence along the track, providing an index of how an animal’s movement behavior changes in space and time based on the autocorrelation of successive movements.

Finally, we add a Naive model for comparison, which always predicts the last observed location for the forecasting task, and a straight line of constant speed filling the gap for imputation. The Naive model estimates the uncertainty based on the spread of past locations.

### Prediction regions

This section describes how base prediction regions are computed. These regions can be further tuned using validation data, yielding equal or better results for all models (Tables S10 and S11).

Let $\textbf{z}$ represent all the input variables (encoder, decoder and static) and $\{t_{i}, i = 1,\dots ,n\}$ the discrete times within the prediction window. TFT predicts quantiles $\hat{x}_{q}(\textbf{z}; t_{i})$ and $\hat{y}_{q}(\textbf{z}; t_{i})$ of the unknown distributions $F_{i}$ of future locations ($X(\textbf{z}; t_{i}), Y(\textbf{z}; t_{i})$), independently for each Mercator coordinate. In what follows, we will simplify our notation by denoting $X\equiv X(\textbf{z}; t_{i})$ and $Y \equiv Y(\textbf{z}; t_{i})$. Thus, the equally-tailed prediction intervals (PIs) for each unknown coordinate and with target coverage error rates (CERs) $\alpha _{x}$ and $\alpha _{y}$ are given by

3 $$\begin{aligned} \text {PI}_{X}= \left\{ x\in [-\pi \text {R}, \pi \text {R}) \mid \hat{x}_{\frac{\alpha _{x}}{2}} \le x \le \hat{x}_{1-\frac{\alpha _{x}}{2}}\right\} , \quad \text {PI}_{Y} = \left\{ y\in \mathbb {R} \mid \hat{y}_{\frac{\alpha _{y}}{2}} \le y \le \hat{y}_{1-\frac{\alpha _{y}}{2}}\right\} \end{aligned}$$

where R is the Earth radius in km. To obtain the PR, we require the actual location (*x*, *y*) to simultaneously lie within both PIs. Hence,

4 $$\begin{aligned} 1 - \alpha _{F} = \mathscr {P}\big ((x,y)\in \text {PR}\big ) = \mathscr {P}(x\in \text {PI}_{X}) \mathscr {P}(y\in \text {PI}_{Y})= (1-\alpha _{X})(1-\alpha _{Y}) \end{aligned}$$

where $\alpha _{F}$ is the family-wise target CER for the PR. Imposing the target error rates of the coordinates to be equal $\alpha _{X}=\alpha _{Y}=\alpha$, we obtain the coordinate error rates associated to a region error rate $\alpha _{F}$:

5 $$\begin{aligned} \alpha = 1 - \sqrt{1-\alpha _{F}} \end{aligned}$$

During training, the model minimizes the total quantile loss (Eq. 15) for estimating the quantiles $\{\hat{x}_{\alpha /2},\hat{x}_{1-\alpha /2}, \hat{y}_{\alpha /2}, \hat{y}_{1-\alpha /2}\}$ of the unknown distribution of locations $F_i$ for the time step $t_i$, with $\alpha$ given by (Eq. 5) and $\alpha _{F} \in \{0.05, 0.1, 0.5\}$. Finally, the PR is obtained by stacking the PIs (Eq. 3):

6 $$\begin{aligned} \text {PR}_{XY} = \text {PI}_{X} \otimes \text {PI}_{Y} \end{aligned}$$

where $\otimes$ denotes the Cartesian product.

For TFT[B], the prediction region corresponds to the highest density region (HDR) of the bivariate distribution of coordinates at the target time step Fi, which contains the target probability 1 - $\alpha _{F}$ within the smallest area. We construct the bivariate distribution from estimates of the univariate distributions, coupled using a Gaussian copula with correlation $\rho$.

For the univariate distributions, we train TFT to predict quantiles $\hat{x}_{q}$ and $\hat{y}_{q}$ (omitting the dependence with $\textbf{z}$ and $t_{i}$), across a uniformly spaced quantile domain with spacing $\textrm{d}q$, treated as a hyperparameter. When $\textrm{d}q$ > 0.01, the quantiles 0.01 and 0.99 are included to preserve information about the distribution tails. Using these quantile predictions as a sample of $F_i$, we estimate the univariate distributions non-parametrically via quantile respectful density estimation. QRDE ensures that the quantiles for the density estimate aligns with those of the input sample. Since the predicted quantiles are uniformly spaced (only approximately for tails when $\textrm{d}q$ > 0.01), the quantile *q* of the sample of quantiles $\{\hat{x}_{q}', q' = dq,\dots , 1-dq\}$ matches the corresponding predicted quantile $\hat{x}_{q}$. Thus, through QRDE we obtain a non-parametric estimate for the univariate distribution that relies solely on the TFT quantile prediction, and that will be as accurate as the predicted quantiles. However, QRDE produces sharp spikes in density at the sample boundaries, which we address with a post-processing step. Specifically, we add synthetic maximum and minimum values to the sample of quantiles by extrapolating the quantile function at the boundaries with a spacing $\textrm{d}q$, and compute the associated QRDE density. The distribution is then restricted to the range of quantiles predicted by TFT, with probabilities rescaled to sum to 1. Extrapolation is performed linearly and locally, using only the three nearest points, and ensuring the probability monotonically decreases at the tails.

For the Gaussian copula modeling the dependence between the univariate distributions, we train a separate TFT to predict the rolling correlation between the coordinates (*X*, *Y*), using centered windows of semi-length 4 days. The output corresponds to the median quantile $\hat{\rho }_{1/2}$, which serves as the predicted correlation, and the endpoints of the 90% CI $\{\hat{\rho }_{0.05}, \hat{\rho }_{0.95}\}$, which quantifies the error in the prediction. We tune the maximum allowed $|\hat{\rho }_{1/2}|$ and the maximum spread of the 90% CI to maximize the prediction region quality in the validation set.

From the bivariate distribution, we estimate the HDR^41^ using two methods. First, we use the standard procedure for a HDR of a bivariate distribution. Let *f*(*x*, *y*) be the density function of the coordinates at the target time step. Then, the $100(1-\alpha _{F})$% HDR is the subset $R(f_{\alpha _{F}})$ of the sample space of (*X*, *Y*) such that

7 $$\begin{aligned} R(f_{\alpha _{F}}) = \{(x,y): f(x,y) \ge f_{\alpha _{F}}\} \end{aligned}$$

where $f_{\alpha _{F}}$ is the largest constant verifying

8 $$\begin{aligned} \mathscr {P}\big ((X,Y) \in R(f_{\alpha _{F}})\big ) \ge 1 - \alpha _{F} \end{aligned}$$

We sample $10^{5}$ observations from the distribution to compute the HDR. The second method uses the convex hull^72^ of the HDR determined by the standard procedure, ensuring the HDR is convex. This approach leads to better results.

SSM estimates for locations at a certain time $t_{i}$ follow a Bivariate Gaussian $\mathscr {N}(\hat{\varvec{\mu }}, \hat{\Sigma }; t_i)$ with mean $\hat{\varvec{\mu }}=(\hat{\mu }_{X}(t_i), \hat{\mu }_{Y}(t_i)$, standard errors $\hat{\sigma }_{X}(t_i)$, $\hat{\sigma }_{Y}(t_i)$ and no correlation. The PR is the HDR encompassing a probability 1 - $\alpha _{F}$ with minimal area, corresponding to the ellipse

9 $$\begin{aligned} (\textbf{w} - \varvec{\hat{\mu }})^T \hat{\Sigma }^{-1} (\textbf{w} - \varvec{\hat{\mu }}) = \chi ^2_{2, \alpha _{F}}, \quad \textbf{w}=\begin{pmatrix}x (t_{i})\\ y(t_{i})\end{pmatrix} \end{aligned}$$

where $\chi ^2_{2, \alpha _{F}}$ is the $\alpha _{F}$ quantile of the chi-squared distribution with 2 degrees of freedom. In our case,

10 $$\begin{aligned} \hat{\Sigma } = \begin{pmatrix} \hat{\sigma }_{X}^{2}(t_{i}) & 0 \\ 0 & \hat{\sigma }_Y^2(t_{i}) \end{pmatrix}\quad \implies \quad \hat{\Sigma }^{-1} = \begin{pmatrix} \hat{\sigma }_X^{-2}(t_i) & 0 \\ 0 & \hat{\sigma }_Y^{-2}(t_i) \end{pmatrix} \end{aligned}$$

which substituted back in (Eq. 9) gives

11 $$\begin{aligned} \frac{(x(t_{i}) - \hat{\mu }_{X}(t_{i}))^2}{\hat{\sigma }_X^2(t_i)} + \frac{(y(t_i) - \hat{\mu }_{Y}(t_i))^2}{\hat{\sigma }_Y^2(t_i)} = \chi ^2_{2, \alpha _{F}} \end{aligned}$$

Thus, the PR is an ellipse centered at $(\hat{\mu }_{X}(t_i), \hat{\mu }_{Y}(t_i)$ with semi-axes $\hat{\sigma }_{X}(t_i)\sqrt{\chi ^2_{2, \alpha _{F}}}$ and $\hat{\sigma }_{Y}(t_i)\sqrt{\chi ^2_{2, \alpha _{F}}}$.

Similar to TFT, the Naive model estimates a PI for each coordinate independently, and constructs the PR with target CER $\alpha _{F}$ by stacking both PI (Eq. 6) with individual target CERs $\alpha$ given by (Eq. 5). For a time $t_{i}$ separated *i* steps from the last observed location, the Naive model estimates the PI as the CI with confidence level 1 - $\alpha$ for the difference between all observed values separated by the same number of steps:

12 $$\begin{aligned} \text {PI}_{k}(t_{i}) = \{\Delta k^{(i)}\in \mathbb {R} \mid \Delta k^{(i)}_{\frac{\alpha }{2}} \le \Delta k^{(i)} \le \Delta k^{(i)}_{1-\frac{\alpha }{2}}\}, \quad k\in \{x,y\} \end{aligned}$$

with

13 $$\begin{aligned} \Delta k^{(i)}=\{\Delta k^{(i)}_{j}=k(t_{j}) - k(t_{j-i}) \mid j\in \mathscr {J}\} \end{aligned}$$

where $\mathscr {J} = \{-M+i,\dots , 0\}$ for the forecasting task and the first half of the imputation time window $i \le \lfloor n/2\rfloor$, $\mathscr {J} = \{n+1+i,\dots , N\}$ for the second half of the imputation time window and $-M$ and *N* are the time steps for the initial and final location, respectively.

### Point prediction

For TFT and TFT[B], the point prediction at time $t_{i}$ is the estimation of the marginal medians ($\hat{x}_{1/2}(\textbf{z}; t_i), \hat{y}_{1/2}(\textbf{z}; t_i)$) of the unknown distribution $F_i$ of the location ($X(\textbf{z}; t_i), Y(\textbf{z}; t_i)$). It is optimized using the quantile loss (Eq. 17) with $q = 1/2$.

For SSMs, it corresponds to the mean $\hat{\varvec{\mu }}=(\hat{\mu }_{X}(t_i), \hat{\mu }_{Y}(t_i)$ of the estimated Bivariate Gaussian $\mathscr {N}(\hat{\varvec{\mu }}, \hat{\Sigma }; t_i)$ associated with time $t_{i}$.

Lastly, the naive model predicts the last observed location ($x(t_0), y(t_0)$) for the forecasting task, while for the imputation task it predicts a straight line between ($x(t_0), y(t_0)$) and the first observed future location ($x(t_{n+1}), y(t_{n+1})$), with constant speed

14 $$\begin{aligned} v=\frac{d\big ((x(t_0), y(t_0)), (x(t_{n+1}), y(t_{n+1})\big )}{T+\Delta t} \end{aligned}$$

where $\Delta t = 6$ hours is the time step between consecutive points, $T = n\Delta t=7$ days is the time span of the imputation window and *d* denotes the great circle distance (Eq. 23).

### Fitting pipeline

#### Hyperparameter tuning

For TFT and TFT[B], we utilized the Optuna^73^ Python package, which employs Bayesian optimization to guide the hyperparameter search. The total number of iterations is 150. The objective function is the minimization of the validation loss while training for a maximum of 30 reduced epochs, containing 60 training batches with batch size 128. The training batches are sequentially selected from the training set, avoiding the repetition of trajectories between consecutive reduced epochs. We report the validation loss for every epoch and use ‘SuccessiveHalvingPruner’ to terminate underperforming trials before completion, allowing the allocation of more resources to more promising ones. All parameters are determined using Optuna, except for the learning rate, which uses the tuner.lr_find method from pytorch_lightning’s Trainer class and falls back to Optuna in case any error is raised. We train TFT on the 5 best hyperparameter sets and select the model with highest prediction region quality *Q* (Eq. 31) on the validation set. Details on the search space, strategies, and optimal values are provided in Table S12.

#### Model training

We train the model in sub-sequences of variable length between half and the maximum specified input days with time step $\Delta t = 6$ hours, and evaluate them in sub-sequences the model has not yet seen, with variable prediction length between 6 hours and 7 days. The length of the prediction window for the validation and test sets is always 7 days, and sequences do not overlap with each other. The maximum number of training epochs is 200, stopping earlier if the validation loss does not improve for 40 consecutive epochs. we fully train TFT on the 5 best performing sets of hyperparameters, and select the model with highest prediction region quality (Eq. 32) on the validation set. For both forecasting and imputation, the optimal number of input days is 4 (Table S12). The optimization objective is the minimization of the total quantile loss

15 $$\begin{aligned} \mathscr {L} = \sum _{k\in \{x,y\}} \sum _{q \in \mathscr {Q}} \sum _{i=1}^{n} \sum _{j=1}^{T} \mathscr {L}_q\big (k_{ij}, \hat{k}_{ij,q}\big ) \end{aligned}$$

where *T* is the number of trajectories, *n* is the number of steps in the prediction window, (*x*, *y*) are the Mercator projections (Eq. 1),

16 $$\begin{aligned} \mathscr {Q}=\Big \{\frac{1}{2}\Big \} \cup \bigcup _{\alpha \in A}\Big \{\frac{\alpha }{2}, 1-\frac{\alpha }{2}\Big \} \end{aligned}$$

is the set of target quantiles for the individual coordinates, where 1/2 (median) accounts for the point prediction and $A = \big \{g(\alpha _{F}) | \alpha _{F} \in \{0.5, 0.1, 0.05\}\big \}$ is the set of target CERs for the individual coordinates, with $g(\alpha _{F})$ given by (Eq. 5), and

17 $$\begin{aligned} \mathscr {L}_q\big (k_{ij}, \hat{k}_{ij,q}\big ) = \max \Big (q \big (k_{ij} - \hat{k}_{ij,q}\big ),\ (1 - q) \big ( \hat{k}_{ij,q} - k_{ij}\big )\Big ) \end{aligned}$$

is the quantile loss between the real coordinate $k_{ij}$ and the predicted *q* quantile $\hat{k}_{ij,q}$ for trajectory *j* at time $t_{i}$.

Training is best performed using a GPU, with a NVIDIA RTX 3090 completing the process in approximately 6–10 hours for forecasting and 9–16 hours for imputation. Inference, however, requires significantly fewer resources and can be performed on a single CPU core, taking less than a second per instance.

#### Prediction region tuning

The TFT model is trained to predict quantiles of individual coordinates. While this should be reflected in the determination of prediction regions, the model is not explicitly optimized for this purpose. Therefore, we introduce an additional optimization step to regulate the size of the PRs while maintaining their proportions. This step minimizes the absolute difference between the target coverage and average of empirical coverages on the validation set. We apply this optimization to all models, resulting in equal or improved performance on the test set (Tables S10 and S11).

Let $\textrm{PR}_{ij}(\alpha _{F})$ be a prediction region for trajectory j at time step $t_{i}$ with target CER $\alpha _{F}$, defined by positive tail lengths $\big \{\hat{\delta }^{x^{-}}_{ij}(\alpha _{F}), \hat{\delta }^{x^{+}}_{ij}(\alpha _{F}), \hat{\delta }^{y^{-}}_{ij}(\alpha _{F}), \hat{\delta }^{y^{+}}_{ij}(\alpha _{F})\big \}$, a central location ($\hat{x}_{ij}(\alpha _{F}),\hat{y}_{ij}(\alpha _{F})$, and a rectangular (TFT, Naive) or elliptical (SSMs) shape. For the rectangular shape:

18 $$\begin{aligned} \text {PR}_{ij}(\alpha _{ F}) = \big [\hat{x}_{ij}(\alpha _{ F}) - \hat{\delta }_{ij}^{x^{-}}(\alpha _{ F}), \hat{x}_{ij}(\alpha _{ F}) + \hat{\delta }_{ij}^{x^{+}}(\alpha _{ F})\big ] \times \big [\hat{y}_{ij}(\alpha _{ F}) -\hat{\delta }_{ij}^{y^{-}}(\alpha _{ F}), \hat{y}_{ij}(\alpha _{ F}) +\hat{\delta }_{ij}^{y^{+}}(\alpha _{ F})\big ] \end{aligned}$$

while for the elliptical shape $\hat{\delta }_{ij}^{x^{\pm }}(\alpha _{ F})=\hat{\delta }_{ij}^{x}(\alpha _{ F})$ and $\hat{\delta }_{ij}^{y^{\pm }}(\alpha _{ F})=\hat{\delta }_{ij}^{y}(\alpha _{ F})$ due to symmetry, and

19 $$\begin{aligned} \text {PR}_{ij}(\alpha _{F}) = \Big \{(x,y)\in [-\pi \text {R}, \pi \text {R}]\times \mathbb {R} \mid \Big (\frac{x-\hat{x}_{ij}(\alpha _{F})}{\hat{\delta }_{ij}^{x}(\alpha _{F})}\Big )^{2} + \Big (\frac{y-\hat{y}_{ij}(\alpha _{F})}{\hat{\delta }_{ij}^{y}(\alpha _{F})}\Big )^{2} \le 1 \Big \} \end{aligned}$$

where R is the Earth radius. Let $\{ {\text{PR}}^{{\prime }} _{{ij}} (\alpha _{F} ){\mid }i \in \{ 1, \ldots ,n\} ,j \in \{ 1, \ldots ,T\}$ denote the set of modified prediction regions, such that $\textrm{PR}_{ij}(\alpha _{F})$ and $\textrm{PR}'_{ij}(\alpha _{F})$ have equal proportions and different area, where *T* is the number of trajectories and *n* is the last time step within the prediction window. We obtain the modified PRs with target CER $\alpha _{F}$ by multiplying all tail lengths by the same parameter $\lambda _{\alpha _{F}}\in \mathbb {R}^{+}$:

20 $$\begin{aligned} \hat{\delta }_{ij}^{\prime k^{\pm }}(\alpha _{F}) = \lambda _{\alpha _{F}}\ \hat{\delta }_{ij}^{k^{\pm }}(\alpha _{F}), \quad k\in \{x,y\}, \ i\in \{1,\dots ,n\}, \ j\in \{1,\dots ,T\} \end{aligned}$$

The optimal value $\lambda ^{*}_{\alpha _{F}}$ minimizes the absolute difference between $\alpha _{F}$ and the average of the empirical CERs $\langle \hat{\alpha }'_{F}(t_i)\rangle$ across time steps for the modified prediction regions on the validation set:

21 $$\begin{aligned} \lambda ^{*}_{\alpha _{F}} = \arg \min _{\lambda _{\alpha _{F}}} \left| \alpha _{F} - \frac{1}{n} \sum _{i=1}^{n} \hat{\alpha }'_{F}(t_i) \right| \end{aligned}$$

where $\hat{\alpha }'_{F}(t_i)$ can be obtained from (Eq. 24) by substituting the original $\textrm{PR}_{ij}(\alpha _{F})$ by their modified counterparts $\textrm{PR}^{\prime}_{ij}(\alpha _{F})$.

For the PRs corresponding to the highest density region of the bivariate distribution, we adjust the target probability of the HDR to match the desired CER. If the maximum probability $p_{\max }$ does not suffice, we expand the boundary of the HDR while preserving its shape, similar to other cases. Let $\hat{\textbf{x}_{ijl}} = (\hat{x}_{ijl}, \hat{y}_{ijl})$ denote the coordinates for the vertex *l* of the HDR ($p = 1$) with target coverage 1 - $\alpha _{F}$. The expanded coordinates are given by

22 $$\begin{aligned} \hat{\textbf{x}'_{ijl}} = \hat{\textbf{x}_{ijl}} + \lambda _{\alpha _{F}} (\hat{\textbf{x}_{ijl}} - \hat{\varvec{\mu }}_{\textbf{ij}}) \end{aligned}$$

where $\hat{\varvec{\mu }}_{\textbf{ij}}$ represents the mean coordinates of the original PR, and $\lambda _{\alpha _{F}}\in \mathbb {R}^{+}$ is the expansion factor. In the implementation we fit four hyperparameters in the validation set:

1. $p_{\max }$: Threshold upon which PR expansion is given by (Eq. 20). Below the threshold, the expansion is computed by taking a larger target probability.
2. $\eta _{\alpha _{F}}$: the target probability if $\eta _{\alpha _{F}} \le 1$, or used to compute the expansion factor otherwise: $\lambda _{\alpha _{F}} = \eta _{\alpha _{F}} - 1$.
3. $\rho _{\alpha _{F},\max }$: maximum absolute correlation. Correlations larger in absolute value than the limit are clipped to the maximum value, respecting the sign.
4. $\Delta \rho _{\alpha _{F},90}$: maximum 90% CI spread. Correlations with uncertainty beyond $\Delta \rho _{\alpha _{F},90}$ are set to zero.

### Evaluation metrics

#### Point prediction: distance error

Given a real location ($x_{ij}, y_{ij}$) and a predicted location ($\hat{x}_{ij}, \hat{y}_{ij}$) at time $t_{i}$ for trajectory *j*, the error in the point prediction is the shortest distance between both points, i.e. the great circle distance

23 $$\begin{aligned} d_{ij} = 2\text {R}\arcsin \sqrt{\sin ^{2}\Big (\frac{\hat{\tilde{\theta }}_{ij} - \tilde{\theta }_{ij}}{2}\Big ) + \cos \tilde{\theta }_{ij}\cos \hat{\tilde{\theta }}_{ij} \sin ^{2}\Big (\frac{\hat{\phi }_{ij} - \phi _{ij}}{2}\Big )} \end{aligned}$$

where $\tilde{\theta }$ and $\phi$ denote latitude and longitude, obtained via the inverse transformation of (Eq. 1), and R is the Earth radius.

#### Prediction region: coverage

The average coverage of the prediction regions with target CER $\alpha _{F}$ at time $t_{i}$ across all trajectories $j \in [1, T]$, $\{\textrm{PR}_{ij}(\alpha _{F}), j = 1,\dots , T\}$, is the proportion of the real locations $\{(x_{ij}, y_{ij}), j = 1,\dots , T\}$ that fall within their respective prediction regions, i.e.

24 $$\begin{aligned} 1 - \hat{\alpha }_{F}(t_i) = \frac{1}{T} \sum _{j=1}^{T} \mathbb {I} \big ( (x_{ij}, y_{ij}) \in \text {PR}_{ij}(\alpha _{F}) \big ) \end{aligned}$$

where $\mathbb {I}(\cdot )$ is the indicator function that equals 1 if the condition inside is true and 0 otherwise, and $\hat{\alpha }_{F}(t_i)$ is the empirical CER (the rate of false positives) at $t_{i}$.

#### Prediction region: area

The area of a surface patch $\mathscr {S}$ on the Earth surface is given by $A = \Omega \text {R}^{2}$, where R is the Earth radius and $\Omega$ is the solid angle

25 $$\begin{aligned} \Omega = \iint _{\mathscr {S}}\sin \theta \ \text {d}\theta \text {d}\phi \end{aligned}$$

being $\theta$ the polar angle and $\phi$ the longitude. From the Mercator projections (Eq. 1), we obtain

26 $$\begin{aligned} \phi = \frac{x}{\text {R}}, \quad \theta = \pi - 2\arctan \big (\exp (y/\text {R})\big ) \end{aligned}$$

Since all models use the Mercator projections, we apply the change of variables $(\theta , \phi ) \rightarrow (x, y)$ on (Eq. 25). Rewriting the integrand in terms of the Mercator coordinates using the relation (Eq. 26) and substituting the Jacobian

27 $$\begin{aligned} J(x,y) = \left| \begin{array}{cc} \partial _x \theta & \partial _y \theta \\ \partial _x \phi & \partial _y \phi \end{array}\right| = \frac{2}{\text {R}^{2}} \frac{\exp (y/\text {R})}{\exp (2y/\text {R})} \end{aligned}$$

we obtain

28 $$\begin{aligned} \Omega = \frac{4}{\text {R}^{2}} \iint _{\mathscr {S}} \frac{\exp (2y/\text {R})}{\big (1 + \exp (2y/\text {R})\big )^{2}}\ \text {d}x \text {d}y \end{aligned}$$

For the rectangle PR $\mathscr {S}_{\text {r}} = \{(x,y) | x_0 \le x \le x_1 \wedge y_0 \le y \le y_1\}$, used in TFT and Naive, we get

29 $$\begin{aligned} \Omega = -\frac{2}{R}(x_{1}-x_{0})\Big [ \frac{1}{1+\exp (2y_{1}/R)} - \frac{1}{1+\exp (2y_{0}/R)}\Big ] \end{aligned}$$

while for the ellipse PR $\mathscr {S}_{\text {e}}=\{(x,y) \mid \big (\frac{x-\mu _x}{a}\big )^{2} + \big (\frac{y-\mu _y}{b}\big )^{2} \le 1 \}$, used in the SSMs, (Eq. 28) can be written as

30 $$\begin{aligned} \Omega = \frac{8a}{\text {R}^{2}}\int _{\mu _{y}-b}^{\mu _y + b} \sqrt{1-\Big (\frac{y-\mu _y}{b}\Big )^{2}} \frac{\exp (2y/\text {R})}{1+\exp (2y/\text {R})} \text {d}y \end{aligned}$$

We compute (Eq. 30) numerically using Simpson’s 3/8 rule. The process starts with $s = 24$ sub-intervals, which doubles until the absolute relative difference between two consecutive iterations is smaller than $10^{-5}$.

For the PR corresponding to the HDR of the bivariate density distribution, we integrate (Eq. 28) over the conforming Delaunay triangulation of the HDR polygon using 3-point Gaussian quadrature. Each triangle is recursively subdivided into four triangles until the absolute relative difference between the integral over the triangle and the sum of the integrals over the sub-triangles is smaller than $10^{-5}$.

#### Prediction region: quality

We define the quality score *Q* for a PR taking into account that an optimal PR should provide an empirical CER $\hat{\alpha }_{F}$ lower or equal to the target CER $\alpha _{F}$, while minimizing the prediction region area. We divide our quality score *Q* into a coverage error rate term $Q_{\alpha }$ and an area term $Q_A$, and use the following criteria for determining their explicit forms: (i) The metric is ranged from 0 (worst) to 1 (best). (ii) The score is truncated on both ends. It is maximum for all values smaller than a reference value, and minimum for all values greater than a maximum value. (iii) Each component penalizes errors in a multiplicative fashion. This is, for a multiplicative factor *r*, the decrease in quality is proportional to the number of multiplications by *r*. (iv) The total quality is obtained as a product of the components:

31 $$\begin{aligned} Q(\hat{\alpha }, A; \alpha , A_{\text {ref}}, A_{\max }) = Q_{\alpha }(\hat{\alpha }; \alpha ) \cdot Q_{A}(A; A_{\text {ref}}, A_{\max }) \end{aligned}$$

The product ensures that poor quality in one variable can not be compensated by a large quality in the other one.

As an example, criterion (i) implies that the drop in quality associated with an increase in the order of magnitude of the area is constant: $\Delta \xi _{A\rightarrow 10A}(A) = \xi (10A_{\text {ref}}$, or that successive doublings of the empirical error rate produce the same quality drop: $\Delta \xi _{\hat{\alpha }\rightarrow 2^{k}\hat{\alpha }} (\hat{\alpha }) = k\xi (2\alpha )$. Thus, the general form of the quality metric verifying criteria (i-iii) is a piecewise function where the transition from worst to best is given by the normalized logarithm deviation, relative to the reference value:

32 $$\begin{aligned} Q(x; x_{\text {ref}}, x_{\max }) = 1 - \xi (x; x_{\text {ref}}, x_{\max }), \quad \xi (x; x_{\text {ref}}, x_{\max }) = {\left\{ \begin{array}{ll} 0, & x < x_{\text {ref}}\\ \left| \frac{\log (xx_{\text {ref}}^{-1})}{\log (x_{\max }x_{\text {ref}}^{-1})}\right| , & x_{\text {ref}} \le x \le x_{\max } \\ 1, & x > x_{\max } \end{array}\right. } \end{aligned}$$

where $\xi (x; x_{\text {ref}}, x_{\max })$ is the penalty term. With this form the joint error is analogous to the inclusion-exclusion principle and guarantees criterion (iv):

33 $$\begin{aligned} \xi (\hat{\alpha }, A; \alpha , A_{\text {ref}}, A_{\max }) = \xi _{\alpha }(\hat{\alpha }; \alpha )\ +\ \xi _{A}(A; A_{\text {ref}}, A_{\max })\ -\ \xi _{\alpha }(\hat{\alpha }; \alpha )\xi _{A}(A; A_{\text {ref}}, A_{\max }) \end{aligned}$$

The explicit form for the CER is

34 $$\begin{aligned} Q_{\alpha }(\hat{\alpha }; \alpha ) = {\left\{ \begin{array}{ll}1, & \hat{\alpha } < \alpha \\ 1 - \left| \frac{\log (\hat{\alpha }\alpha ^{-1})}{\log \alpha }\right| , & \alpha \le \hat{\alpha } \le 1\end{array}\right. } \end{aligned}$$

while for the area term:

35 $$\begin{aligned} Q_{A}(A; A_{\text {ref}}, A_{\max }) = {\left\{ \begin{array}{ll} 1, & A < A_{\text {ref}}\\ 1 - \left| \frac{\log (AA_{\text {ref}}^{-1})}{\log (A_{\max }A_{\text {ref}}^{-1})}\right| , & A_{\text {ref}} \le A \le A_{\max } \\ 0, & A > A_{\max } \end{array}\right. } \end{aligned}$$

For the forecasting task, the area $A_{\max }$ is the maximum area spanned by a southern elephant seal moving at maximum speed all the time, with an unknown directionality:

36 $$\begin{aligned} \text {A}_{\max }(t_i) = \pi \big (i\Delta t v_{\max }\big )^{2} \end{aligned}$$

where *i* is the time index, $\Delta t = 6$ hours is the time step size and $v_{\max }=10$ km/h is the maximum speed for a southern elephant seal. Thus, it is the area of a circle with radius equal to the maximum distance the animal would travel, assuming it could keep its maximum speed indefinitely. For the imputation task, we add the constraint that the animal should arrive at the first observed future location at time $t_{n+1}$. The region of maximal area is given by the intersection of the circle (Eq. 36) centered at the starting location ($x_0, y_0$) with radius $r(t_i) = i\Delta tv_{\max }$, and the circle centered at the final location ($x_{n+1}, y_{n+1}$) with radius given by the remaining time for arrival $R(t_i) = (n+1-i)\Delta tv_{\max }$:

37 $$\begin{aligned} A_{\scriptscriptstyle \max } = R^2 \cos ^{-1}\left( \frac{d^2 + R^2 - r^2}{2dR} \right) + r^2 \cos ^{-1}\left( \frac{d^2 + r^2 - R^2}{2dr} \right) - \frac{1}{2} \sqrt{(-d + R + r)(d + R - r)(d - R + r)(d + R + r)} \end{aligned}$$

where *d* is the great circle distance (Eq. 23) between ($x_0, y_0$) and ($x_{n+1}, y_{n+1}$).

For the forecasting task, we define the reference area $A_{\text {ref}}(\alpha _F, t_i)$ as the area of the confidence region with target CER $\alpha _{F}$ for the location at time $t_{i}$. We compute the region using percentile bootstrap with $10^4$ replications. Each replication corresponds to a trajectory where displacements are sampled with replacement from the empirical distribution of displacements $\{\Delta k(t_i)\}$ within the prediction window:

38 $$\begin{aligned} k^{*}(t_i) = k(t_{0}) + \sum _{s=1}^{i} \Delta k^{*(s)}, \quad k\in \{x,y\} \end{aligned}$$

where $\Delta k^{*(s)}$ denotes the *s*-th resampled displacement for the bootstrapped trajectory. This way, the reference area will scale with the target CER and with the animal movement: it will be lower for animals that tend to stay still, and larger for animals moving greater distances; as well as lower for ballistic movement compared to random movement patterns.

For the imputation task, the endpoints of the bootstrapped trajectories must coincide with the observed locations at both sides of the gap. Thus, we obtain the coordinates for each bootstrap replication $k^{*}(t_i)$ by permuting the ordering of the displacements $\{\Delta k(t_i)\}$, such that

39 $$\begin{aligned} k^{*}(t_i) = k(t_{0}) + \sum _{s=1}^{i} \Delta k(t_{\sigma ^{*}(s)}) \end{aligned}$$

where $\sigma ^{*}: \{1,\dots , n\} \rightarrow \{1,\dots , n\}$ is the permutation applied to the time indices within the imputation window.

### Interpretation

#### Feature importance

We estimate the feature importance as the output of the variable selection networks from TFT, which compute selection weights for each feature at every time step. These weights are generated by passing the transformed input and an external context vector through a gated residual network (GRN) and a softmax layer, ensuring that the selection process is context-dependent and differentiable . The selection weights are used to weight the input features, dynamically adjusting their importance based on the available data for the prediction task. This allows TFT to prioritize different features as necessary, depending on the context of each trajectory. By aggregating the variable selection weights across time steps and trajectories, we estimate the overall importance of each feature for the model output.

#### Temporal importance

TFT uses the transformer architecture, which relies on the attention mechanism. The attention mechanism weighs the importance of different elements in an input sequence when producing an output. These weights are obtained using the softmax function, which ensures that they sum to 1 and can be compared across training instances. Thus, by analyzing the attention weights across time, we can estimate which temporal windows have the highest impact on the model output. In what follows we will describe how to compute these weights.

TFT uses the Temporal Self-Attention Layer to capture long-term dependencies within the trajectories. The underlying mechanism is the Interpretable Multi-Head Attention, which aggregates the attention weights of several heads. Each attention head uses the standard attention mechanism based on vector queries *Q* and keys *K* with dimension $d_k$ and values *V* with dimension $d_v$^38^:

40 $$\begin{aligned} \text {Attention}(Q, K, V)= A(Q,K)V, \quad A(Q,K)=\text {softmax}\Big (\frac{QK^{T}}{\sqrt{d_{k}}}\Big ) \end{aligned}$$

where *A*(*Q*, *K*) are the attention weights and $1/\sqrt{d_k}$ is added for numerical stabilization. Queries, keys and values depend on the input embeddings *X*, specific for each trajectory:

41 $$\begin{aligned} Q=X_{p}W_{Q}, \quad K=XW_{K}, \quad V=XW_{V} \end{aligned}$$

where the *W* matrices are weight matrices transforming the input embedding into the corresponding smaller dimension (query-key or value), and the *p* in $X_p$ indicates it only uses the embeddings corresponding to time steps in the prediction time region. *Q*, *K* and *V* can be interpreted as:

- Queries (*Q*): representations for the prediction time steps that act as a set of questions the model uses to seek relevant information from other time steps.
- Keys (*K*): representations of past time steps (and future time steps with known information) that act as a set of answers to the queries.
- Attention weights *A*(*Q*, *K*): The dot product $QK^{\text {T}}$ indicates how relevant is the information from the other time steps to the queries for the prediction time steps. We use causal attention for the forecasting task, which masks (sets to zero) all the attention weights for future and current time steps in the prediction time window. This is, the model can attend to time steps $i'$ in the prediction time window, but only if they precede the target time step: $i' < i, i \in \{1, \dots , n\}$.
- Values (*V*): The information to be aggregated to the embeddings.

The Interpretable Multi-Head Attention aggregates the information of multiple attention heads $h \in \{1, \dots ,n_H\}$, defined by their distinct query and key weight matrices $W_{Q}^{(h)}$, $W_{K}^{(h)}$ and a common value weight matrix $W_V$, with $d_v = d_k = d_{\text {model}}/n_H$, being $d_{\text {model}}$ the dimensionality of the model’s internal representation (hidden state size). The differences in queries and keys enables each head to learn different temporal patterns from the same input sequence, while having a common $W_V$ ensures that attention weights are comparable across heads. By aggregating the multi-head attention of the first prediction time step across trajectories for each past time step, we obtain the relative importance of each past time index in the model output. This can help to identify temporal windows or patterns that have a higher impact in the output.

#### Error analysis

We train gradient boosted tree (GBT) regressors to predict the PR area and distance error of the TFT output, aiming to identify factors influencing model performance. Following the chronological dataset split (Fig. S9a), we train the performance regressors on the training set, tune hyperparameters on the validation set, and evaluate the RMSE on the test set. A lower RMSE, compared to the baseline regressor that always predicts the sample average for the training set, indicates greater confidence in the model’s predictive capabilities. We then compute exact SHAP values^74,75^ for the test set to determine each feature’s impact on the predicted performance metrics.

Input includes trajectory characteristics, such as average, standard deviation and correlations among environmental features; metadata properties (sex, weight, length), number of missing values, Hurst exponent of direction angle, net and cumulative movement on each coordinate, and average speed. We use the minimum redundancy, maximum relevance (mRMR^76^) algorithm on the training set to retain the most informative features and eliminate redundant ones. The inclusion cutoff is based on the minimum mutual information each feature has with the target variable, relative to the most informative feature. This threshold is optimized as a hyperparameter to minimize validation RMSE, along with the GBT hyperparameters.

### Statistical analysis

All the confidence intervals appearing in the main and supplementary tables and figures are computed using bias-corrected and accelerated (BCa) bootstrap with $10^{5}$ iterations, with a confidence level $\alpha = 0.05$.

**Table 1:** Performance metrics for the forecasting task. Sample average and 95% CI between brackets for the point prediction error (first column) and prediction region performance (last three columns) for each model. Arrows indicate whether higher ($\uparrow$) or lower ($\downarrow$) values are better. Underline denotes the top models with indistinguishable performance, while bold highlights the best model at a 95% confidence level. The point prediction error is the shortest distance between the real point and the predicted one, averaged across trajectories and time steps. Prediction performance is assessed using the quality metrics: coverage error rate ($Q_{\alpha }$), area ($Q_{A}$), and overall quality (*Q*), averaged across trajectories, time steps, and target coverage error rates $\alpha _{F} \in \{0.05, 0.1, 0.5\}$. Table S2 displays the areas and coverage error rates that produce the reported quality scores. For comparison, Table S13 presents the quality scores before prediction region tuning, with corresponding areas and coverage error rates shown in Table S14.

	Point prediction	Prediction region		
Model	Distance [km] $\downarrow$	$Q_{\alpha }$ $\uparrow$	$Q_{\text {A}}$ $\uparrow$	*Q* $\uparrow$
TFT[B]	66.4 [59.8, 73.7]	0.96 [0.90, 1.00]	**0.74 [0.73, 0.76]**	**0.72 [0.68, 0.75]**
TFT	67.3 [60.6, 74.6]	0.97 [0.92, 1.00]	0.63 [0.61, 0.66]	0.62 [0.59, 0.65]
TFT[s]	162.9 [121.9, 234.0]	0.76 [0.57, 0.90]	0.38 [0.30, 0.48]	0.26 [0.19, 0.34]
Naive	107.2 [94.7, 121.1]	0.93 [0.87, 0.97]	0.45 [0.42, 0.48]	0.41 [0.39, 0.44]
RW	95.5 [81.8, 110.2]	0.98 [0.95, 0.99]	0.44 [0.39, 0.49]	0.43 [0.39, 0.47]
CRW	188.8 [165.0, 212.9]	0.91 [0.85, 0.96]	0.31 [0.27, 0.34]	0.24 [0.21, 0.28]
MP	80.4 [69.5, 93.6]	0.96 [0.93, 0.99]	0.50 [0.45, 0.55]	0.47 [0.43, 0.52]

**Table 2:** Performance metrics for the imputation task. Sample average and 95% CI between brackets for the point prediction error (first column) and prediction region performance (last three columns) for each model. Arrows indicate whether higher ($\uparrow$) or lower ($\downarrow$) values are better. Underline denotes the top models with indistinguishable performance, while bold highlights the best model at a 95% confidence level. The point prediction error is the shortest distance between the real point and the predicted one, averaged across trajectories and time steps. Prediction performance is assessed using the quality metrics: coverage error rate ($Q_{\alpha }$), area ($Q_{A}$), and overall quality (*Q*), averaged across trajectories, time steps, and target coverage error rates $\alpha _{F} \in \{0.05, 0.1, 0.5\}$. Table S3 displays the areas and coverage error rates that produce the reported quality scores. For comparison, Table S15 presents the quality scores before prediction region tuning, with corresponding areas and coverage error rates shown in Table S16.

	Point prediction	Prediction region		
Model	Distance [km] $\downarrow$	$Q_{\alpha }$ $\uparrow$	$Q_{\text {A}}$ $\uparrow$	*Q* $\uparrow$
TFT[B]	24.1 [22.2, 26.4]	0.96 [0.92, 0.99]	0.87 [0.86, 0.89]	0.84 [0.81, 0.87]
TFT	24.7 [22.7, 27.0]	0.96 [0.92, 0.99]	0.88 [0.87, 0.89]	0.85 [0.82, 0.87]
TFT[s]	122.4 [93.3, 155.4]	0.86 [0.68, 0.98]	0.28 [0.20, 0.39]	0.21 [0.13, 0.32]
Naive	27.5 [25.1, 30.2]	0.97 [0.95, 0.98]	0.36 [0.34, 0.38]	0.34 [0.32, 0.36]
RW	28.8 [27.5, 30.3]	0.96 [0.95, 0.97]	0.77 [0.75, 0.78]	0.73 [0.72, 0.75]
CRW	42.7 [40.8, 45.0]	0.90 [0.88, 0.92]	0.57 [0.55, 0.60]	0.48 [0.46, 0.50]
MP	31.9 [30.4, 34.0]	0.96 [0.95, 0.97]	0.61 [0.60, 0.63]	0.58 [0.56, 0.59]

**Table 3:** Performance metrics for the forecasting task in unseen regions. Sample averages and 95% CIs between brackets for the distance error in location predictions and the quality *Q* of the prediction regions. Both metrics are averaged across trajectories and time steps, while *Q* is further averaged over target coverage errors $\alpha _{F}\in \{0.05,0.1,0.5\}$. Arrows indicate whether higher ($\uparrow$) or lower ($\downarrow$) values are better. Underline denotes the top models with indistinguishable performance, while bold highlights the best model at a 95% confidence level. The middle columns report results for each train–test split: one geographic region (Fig. S10b) is held out as the test set, and the others serve for training and validation. $n_{\text {train}}$ and $n_{\text {test}}$ denote the number of trajectories in the training and test sets. The last column shows the average performance in unseen regions across the five realizations.

Test region	ID	1	2	3	4	5	Average
	$n_{\text {train}}$	400	424	396	423	122	353
	$n_{\text {test}}$	47	12	39	48	372	104
Distance [km]$\downarrow$	TFT[B]	88.0 [76.3, 107.3]	90.2 [64.8, 164.7]	58.8 [50.6, 69.4]	91.1 [82.4, 101.1]	79.3 [75.8, 83.0]	81.5 [73.9, 91.6]
	TFT	86.9 [74.1, 112.1]	88.6 [64.2, 164.0]	61.0 [52.8, 71.3]	79.8 [72.8, 88.2]	92.8 [87.3, 109.3]	81.8 [74.0, 92.2]
	Naive	97.3 [85.3, 110.9]	119.8 [89.1, 188.3]	66.2 [55.6, 79.6]	115 [103, 128]	109 [104, 114]	101.5 [92.8, 112.3]
	RW	96.1 [84.4, 108.6]	96.6 [88.8, 106.2]	84.4 [71.3, 106.7]	134.8 [88.0, 184.2]	92.0 [81.3, 101.9]	100.8 [90.1, 111.5]
	CRW	115.2 [96.4, 160.9]	388 [379, 399]	408 [364, 448]	86.9 [71.0, 100.0]	120 [105, 129]	224 [155, 230]
	MP	93.0 [78.3, 121.2]	85.1 [69.5, 101.3]	49.6 [34.1, 61.5]	82.7 [59.6, 105.6]	95.6 [80.6, 103.2]	81.2 [70.2, 86.7]
*Q* $\uparrow$	TFT[B]	0.47 [0.42, 0.52]	0.52 [0.42, 0.62]	0.60 [0.55, 0.65]	0.48 [0.43, 0.52]	0.48 [0.47, 0.49]	0.51 [0.48, 0.53]
	TFT	0.58 [0.52, 0.63]	0.59 [0.52, 0.66]	0.54 [0.49, 0.58]	0.49 [0.46, 0.53]	0.50 [0.49, 0.52]	0.54 [0.51, 0.56]
	Naive	0.40 [0.36, 0.43]	0.29 [0.26, 0.34]	0.49[0.46, 0.52]	0.30[0.29, 0.32]	0.37 [0.36, 0.38]	0.37 [0.35, 0.38]
	RW	0.43 [0.28, 0.55]	0.28 [0.22, 0.36]	0.49 [0.44, 0.57]	0.42 [0.26, 0.57]	0.47 [0.43, 0.51]	0.42 [0.36, 0.46]
	CRW	0.38 [0.25, 0.48]	0.18 [0.14, 0.23]	0.49 [0.40, 0.59]	0.40 [0.33, 0.48]	0.41 [0.39, 0.44]	0.37 [0.33, 0.40]
	MP	0.39 [0.26, 0.51]	0.28 [0.21, 0.36]	0.60 [0.54, 0.69]	0.54 [0.40, 0.66]	0.53 [0.49, 0.56]	0.47 [0.42, 0.50]

**Table 4:** Performance metrics for the imputation task in unseen regions. Sample averages and 95% CIs between brackets for the distance error in location predictions and the quality *Q* of the prediction regions. Both metrics are averaged across trajectories and time steps, while *Q* is further averaged over target coverage errors $\alpha _{F}\in \{0.05,0.1,0.5\}$. Arrows indicate whether higher ($\uparrow$) or lower ($\downarrow$) values are better. Underline denotes the top models with indistinguishable performance, while bold highlights the best model at a 95% confidence level. The middle columns report results for each train–test split: one geographic region (Fig. S10b) is held out as the test set, and the others serve for training and validation. $n_{\text {train}}$ and $n_{\text {test}}$ denote the number of trajectories in the training and test sets. The last column shows the average performance in unseen regions across the five realizations.

Test region	ID	1	2	3	4	5	Average
	$n_{\text {train}}$	400	424	396	423	122	353
	$n_{\text {test}}$	47	12	39	48	372	104
Distance [km]$\downarrow$	TFT[B]	199 [118, 323]	30.2 [24.4, 41.8]	26.2 [23.5, 29.4]	38.3 [29.2, 79.7]	32.0 [30.9, 33.2]	65.2 [46.7, 86.5]
	TFT	133.1 [83.7, 209.7]	29.8 [24.5, 39.2]	33.5 [29.4, 39.2]	36.7 28.8, 71.9]	33.0 [31.8, 34.3]	53.2 [41.5, 66.6]
	Naive	30.7 [27.7, 34.0]	35.1 [28.8, 46.5]	26.3 [23.5, 29.7]	30.5 [27.9, 33.9]	30.0 [28.9, 31.1]	30.5 [28.7, 32.6]
	RW	36.5 [32.6, 42.3]	34.8 [31.8, 39.1]	34.5 [31.0, 41.6]	28.4 [25.3, 30.5]	38.4 [37.1, 39.8]	34.5 33.0, 36.3]
	CRW	35.4 [32.1, 40.1]	32.3 [30.0, 35.6]	29.2 [26.6, 32.7]	31.9 [27.4, 35.8]	33.8 [32.9, 34.9]	32.5 [31.2, 34.1]
	MP	32.4 [29.3, 36.9]	33.6 [30.7, 37.2]	29.2 [26.8, 32.5]	30.5 [25.7, 34.6]	30.7 [29.8, 31.8]	31.3 [30.0, 32.8]
*Q* $\uparrow$	TFT[B]	0.66 [0.61, 0.70]	0.56 [0.44, 0.66]	0.58 [0.55, 0.62]	0.56 [0.54, 0.59]	0.47 [0.46, 0.49]	0.56 [0.53, 0.58]
	TFT	0.65 [0.60, 0.70]	0.65 [0.56, 0.74]	0.60 [0.58, 0.64]	0.47 [0.43, 0.51]	0.61 [0.60, 0.62]	0.60 [0.57, 0.62]
	Naive	0.35 [0.33, 0.37]	0.23 [0.22, 0.25]	0.37 [0.36, 0.39]	0.24 [0.23, 0.25]	0.27 [0.27, 0.28]	0.29 [0.28, 0.30]
	RW	0.49 [0.39, 0.58]	0.33 [0.26, 0.40]	0.54 [0.45, 0.63]	0.50 [0.38, 0.62]	0.47 [0.44, 0.50]	0.47 [0.43, 0.51]
	CRW	0.56 [0.48, 0.62]	0.30 [0.23, 0.37]	0.55 [0.46, 0.61]	0.49 [0.37, 0.55]	0.57 [0.55, 0.59]	0.49 [0.46, 0.52]
	MP	0.60 [0.51, 0.67]	0.33 [0.26, 0.41]	0.62 [0.54, 0.69]	0.52 [0.40, 0.59]	**0.66** [0.64, 0.67]	0.55 [0.51, 0.58]

## Supplementary Information

Supplementary Information.

## Data Availability

The southern elephant seal data that support the findings of this study are available from Australia’s Integrated Marine Observing System (https://imos.org.au) – IMOS is enabled by the National Collaborative Research Infrastructure Strategy (NCRIS). Environmental variables are available at the Copernicus ERA5 (DOI:10.24381/CDS.ADBB2D47) and ORAS5 (DOI:10.24381/CDS.67E8EEB7) datasets. All the code is available at the github repository: https://github.com/medinajorge/PR_animal_tracking.
