# Supplementary material for: Improving prediction region accuracy in marine animal movement with temporal fusion transformer
Source: Sci Rep. 2025 Dec 4;16:297. doi: 10.1038/s41598-025-29520-2 (PMC12770584; doi:10.1038/s41598-025-29520-2)
Supplement: Supplementary file 1 — Supplementary Information. [file 41598_2025_29520_MOESM1_ESM.pdf]

Supplementary Materials for

Improving Prediction Region Accuracy in Marine Animal Movement with Temporal Fusion Transformer

Jorge Medina Hernández *et al.*

*Corresponding author email: medinahdezjorge@gmail.com

This PDF contains:
Figs. S1 to S12
Tables S1 to S16

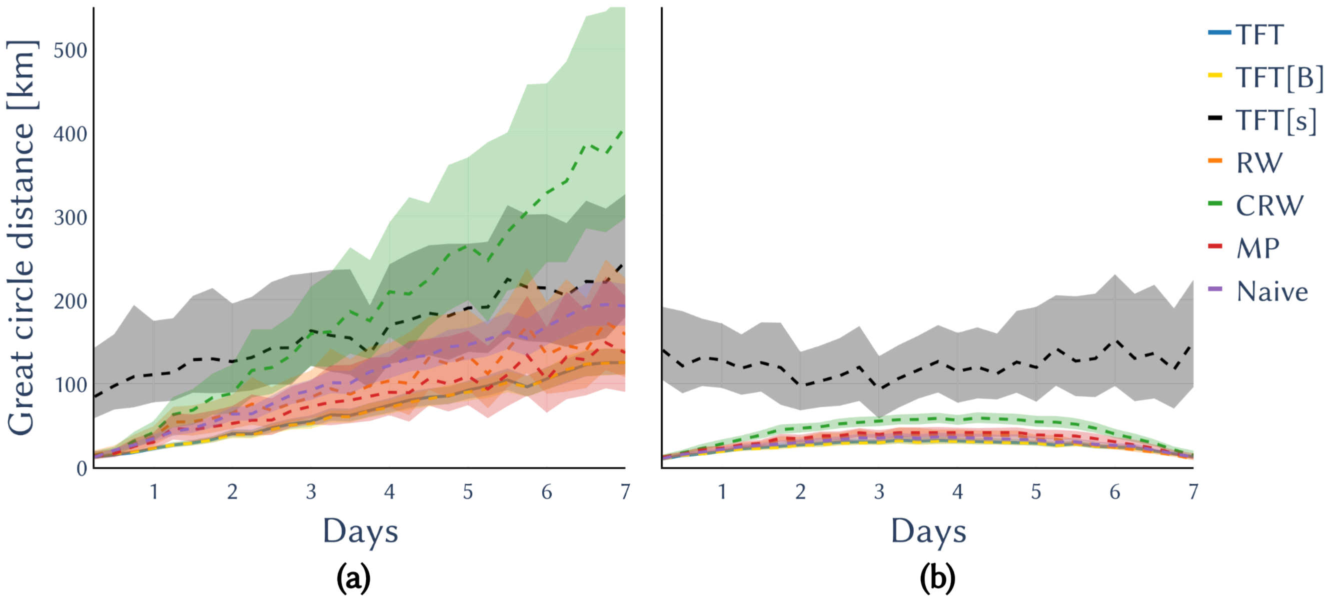

Figure S1. Evolution of the Point Prediction Error. Great circle distance between the predicted and real location over the 7 days within the prediction window, for the forecasting **(a)** and imputation **(b)** tasks. TFT is depicted as a blue solid line when trained on the train dataset, as a dashed yellow line when output quantiles are used to predict the bivariate distribution (TFT[B]), and as a dashed black line when trained on a single trajectory (TFT[s]); SSMs as dashed lines, and the Naive forecaster as violet dots. Bands represent 95% CIs. **(a)**Aside from initially TFT[s] and CRW, which has a larger error, most models have indistinguishable average performance at a given time step, for a confidence level of 0.95. TFT has lower error with statistical significance at any given time step when compared to Naive, CRW and TFT[s]. **(b)**TFT, TFT[B], Naive and Random Walk have mostly indistinguishable average performance at a given time step (confidence = 0.95).

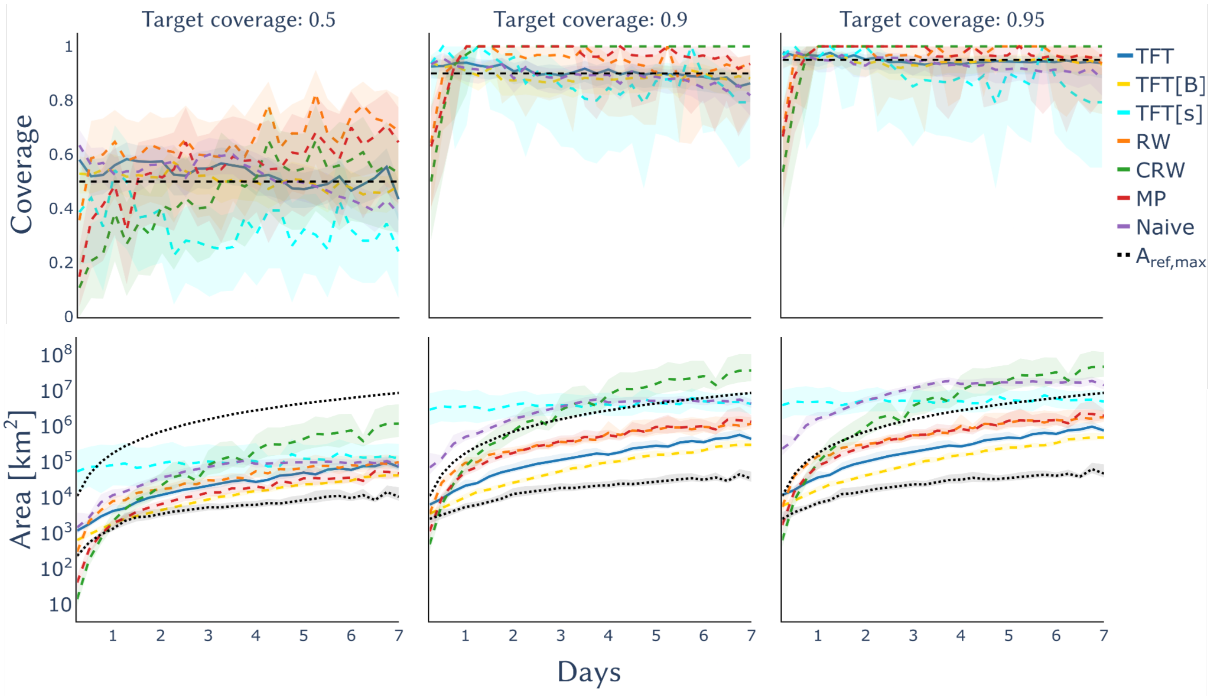

(a)

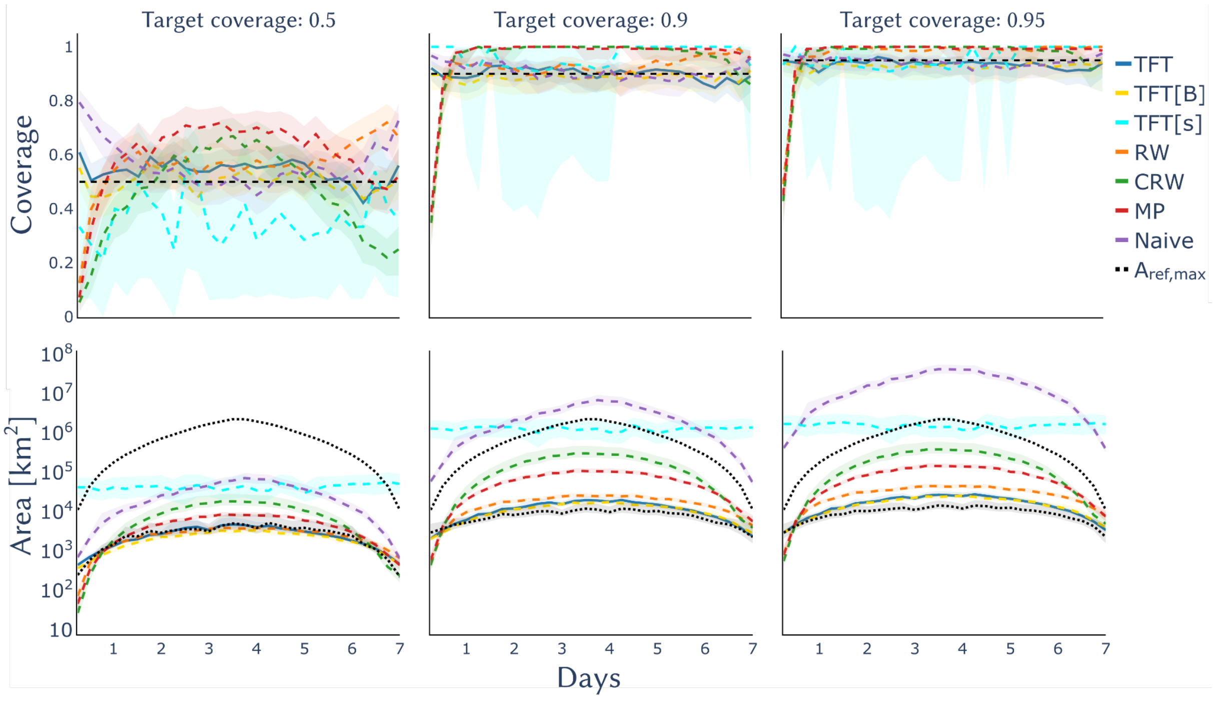

(b)

Figure S2. Evolution of Coverage and Area of the Prediction Region. Coverage (top) and area (bottom) of the PR over 7 future days, for the forecasting (a) and imputation (b) tasks. The target coverages 1 - $\alpha_F \in \{0.5, 0.9, 0.95\}$ and the reference and maximum areas are plotted as black dots for reference. TFT is depicted as a blue solid line when trained on the train dataset, as a dashed yellow line when output quantiles are used to predict the bivariate distribution (TFT[B]), and as a dashed cyan line when trained on a single trajectory (TFT[s]). SSMs and Naive are plotted as dashed lines. Bands represent 95% CIs. While TFT, Naive, RW and MP match the target coverage on average, TFT achieves this using less area.

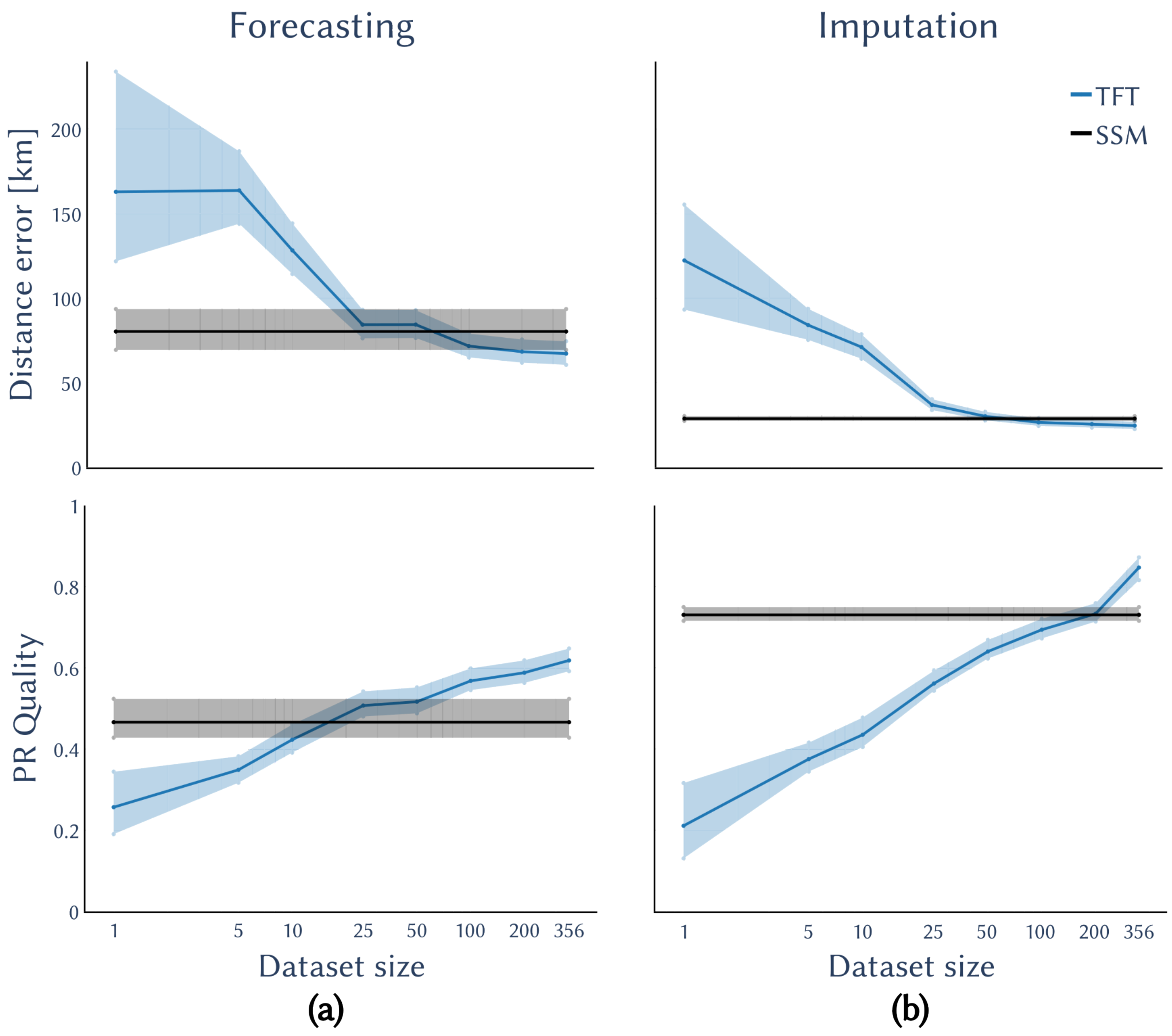

Figure S3. Impact of training dataset size on TFT performance Distance error (top) and prediction region quality (bottom) for forecasting (left) and imputation (right) tasks with the TFT model (blue) trained on datasets ranging from 1 to 356. The best-performing SSM models (MP for forecasting and RW for imputation) are shown in black for comparison. Confidence bands represent 95% confidence intervals. In forecasting, TFT requires n_{train}=25 and n_{train}=10 training trajectories to achieve a performance comparable to the best SSM in terms of distance error and PR quality, and surpasses SSMs in PR quality for training sizes of at least n_{train}=100 trajectories, but not in distance error with statistical significance for $n_{\text{train}} < 356$. In imputation, TFT matches the best SSM performance at n_{train}=200 for PR quality and n_{train}=50 for distance error, and outperforms them in PR quality for n_{train}=300 and in distance error for n_{train}=200.

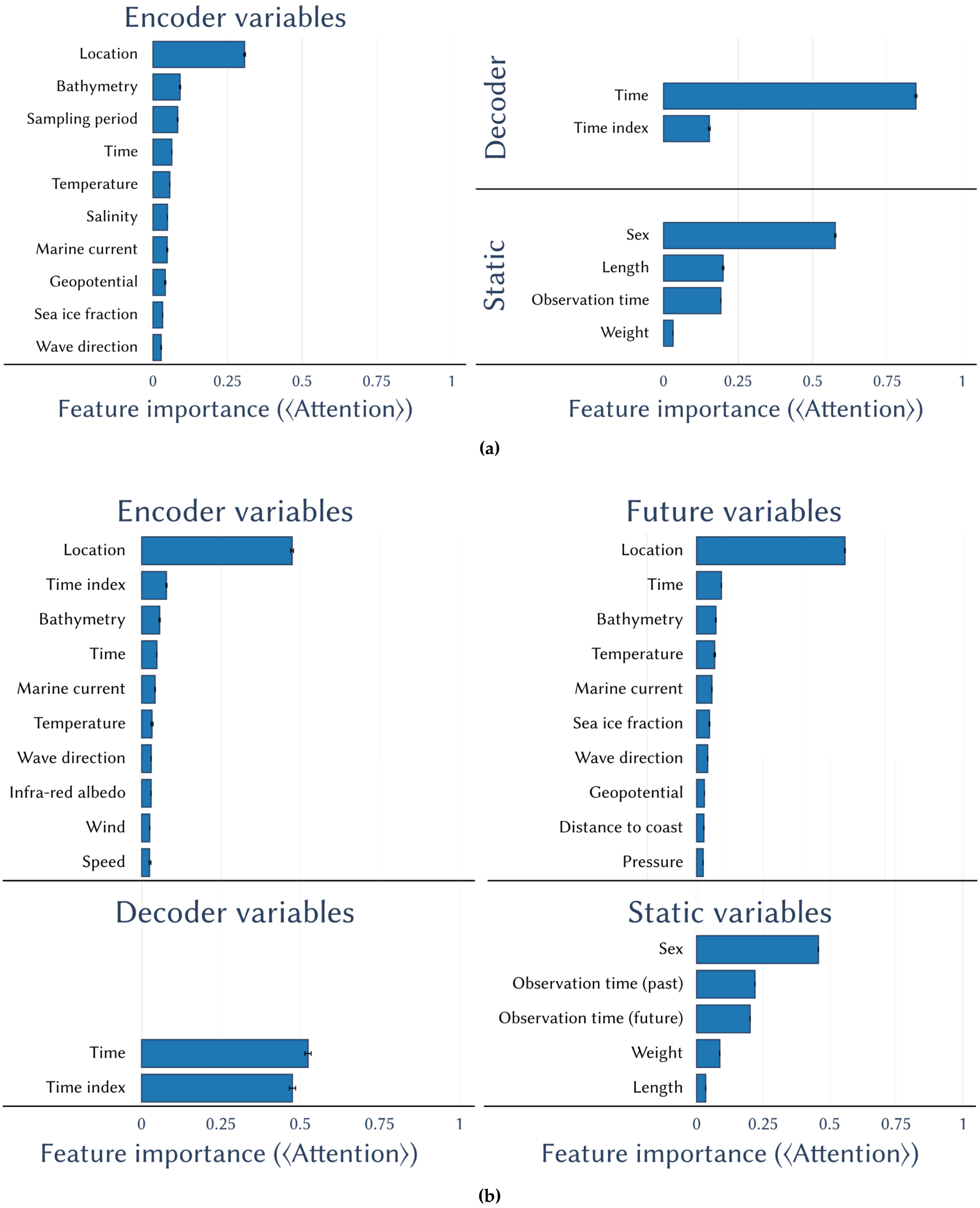

Figure S4. Aggregate feature importances for the Temporal Fusion Model. Average variable selection weights highlighting the most influential features in the output prediction, relative to other variables of the same type, for the forecasting **(a)** and imputation **(b)** tasks. Encoder variables contain known information before the forecasting time window, decoder variables correspond to known variables within the forecasting time window, and static variables are features that do not change over time (metadata). Error bars represent 95% CIs.

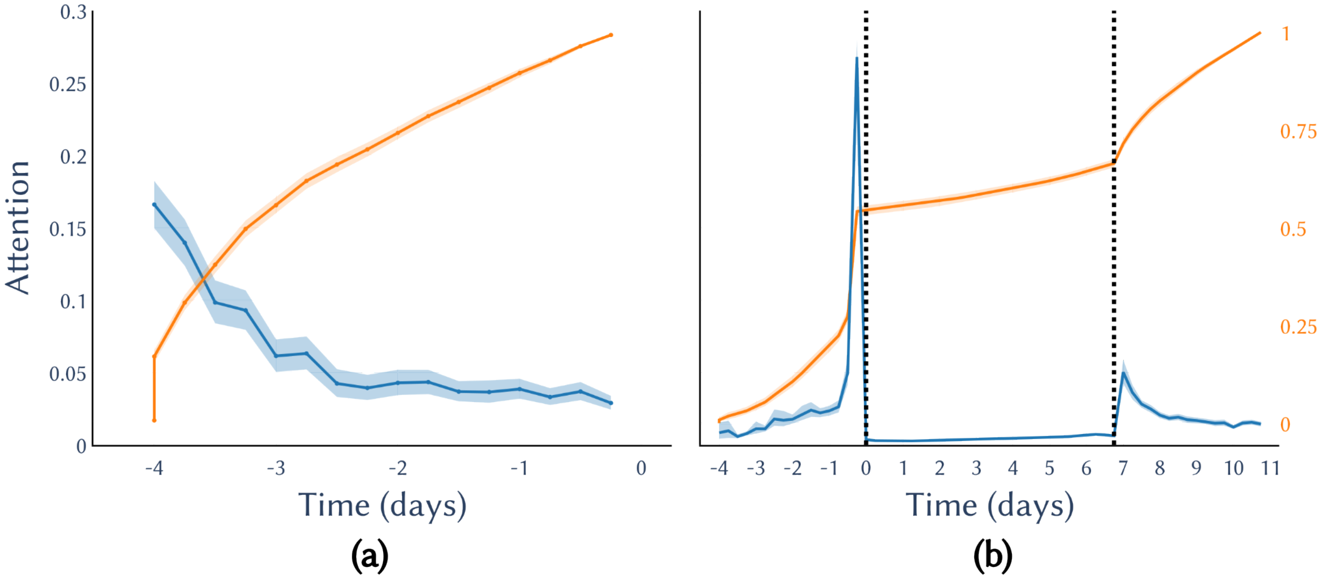

Figure S5. Attention weights across time for the Temporal Fusion Model. Average of the normalized attention weights for each time step (blue), and its cumulative distribution (orange), for the forecasting **(a)** and imputation **(b)** tasks. The prediction region lasts from t= 0 to t= 7 days - 6 hours. The 95% confidence intervals are represented by bands. For the forecasting task, information further from the past is weighted higher, while for the imputation task, the time steps closest to the imputation time window (black dots) are weighted higher, both in the future and past.

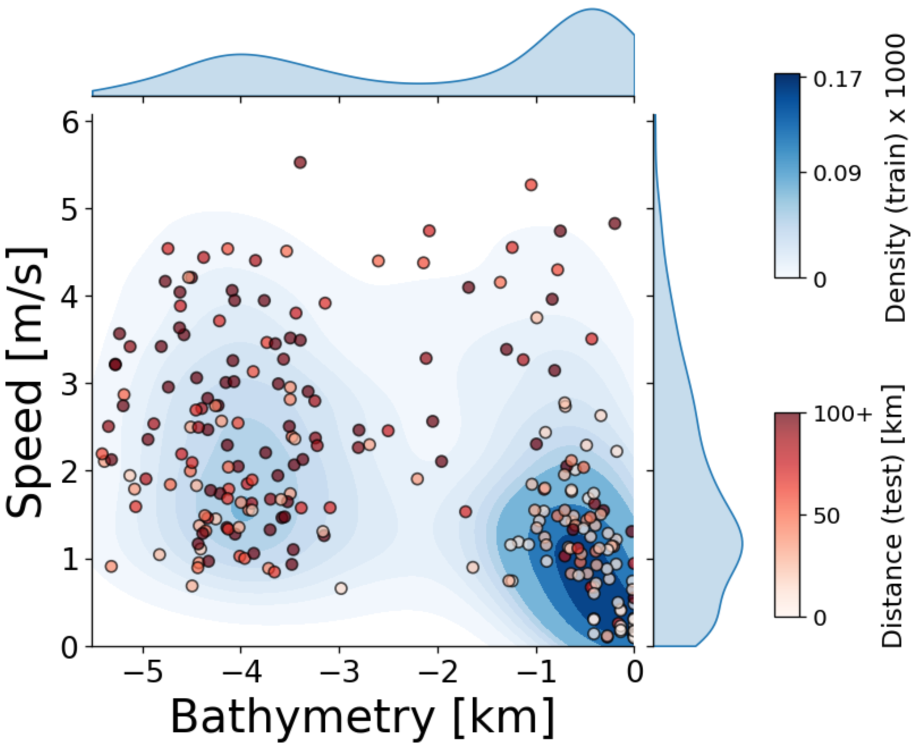

(a)

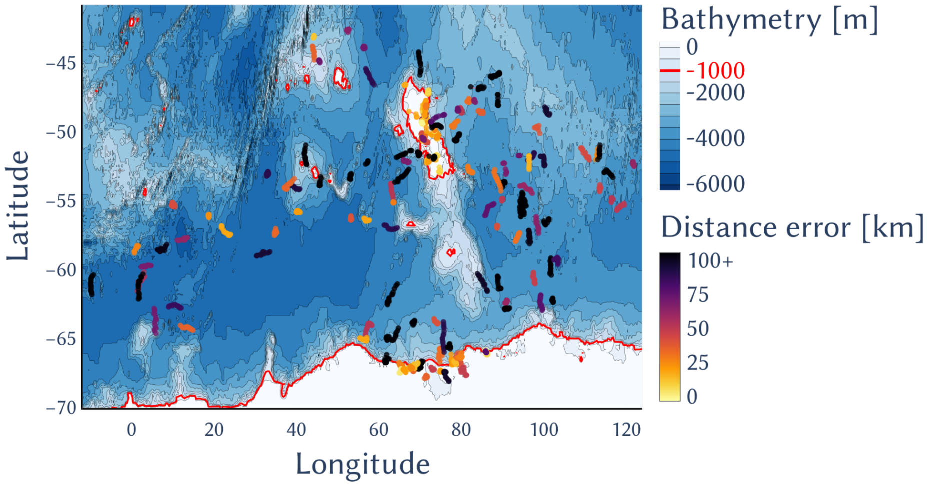

(b)

Figure S6. Predictors of distance error for the Forecasting Task. (a) Test distance errors and density of the training data as a function of speed and bathymetry. Average distance error for each trajectory in the test set as a function of the average speed and bathymetry levels across encoder data points. The distance error is colorscaled from white (no error) to red (>100km). The kernel density estimate of the average bathymetry and speed for the training data is plotted in blue, indicating in which regions there is more training data available. Distance error tends to increase with speed and decrease with bathymetry. The distance error and the training data availability have a Spearman correlation of -0.56. **(b) Location data for trajectories with speed under 3 km/h.** Location coordinates for trajectories with average speeds below 3 km/h across the encoder time window. Points are color-coded by distance error, ranging from yellow (0 km) to black (over 100 km). Bathymetry is shaded from white (shallow) to dark blue (deep), with the -1000 m isoline marked in red. The average distance error is 32 km (C.I. [24, 44] km) within the isoline, compared to 91 km (C.I. [81, 103] km) outside of it.

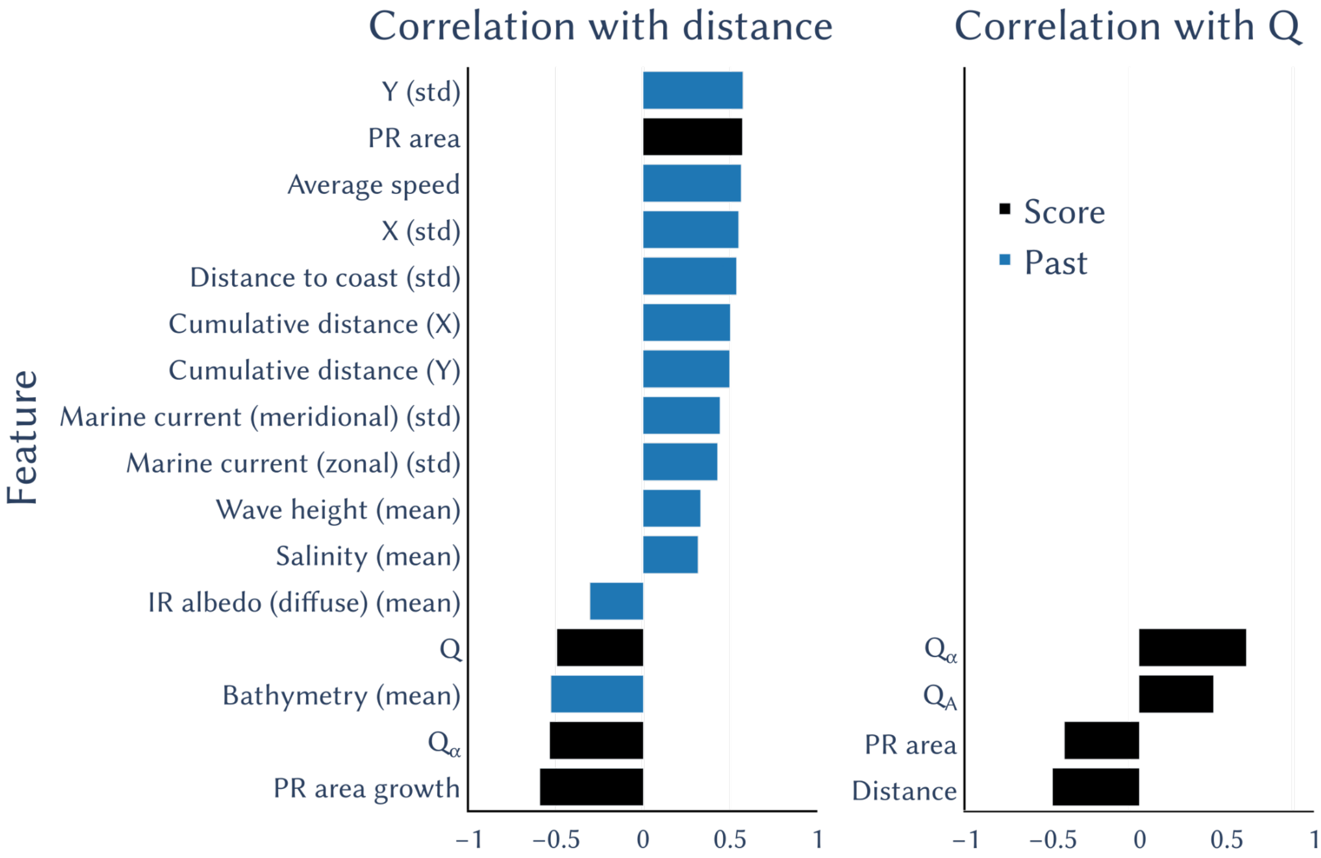
Correlation with distance
Correlation with Q
Feature
Y (std)
PR area
Average speed
X (std)
Distance to coast (std)
Cumulative distance (X)
Cumulative distance (Y)
Marine current (meridional) (std)
Marine current (zonal) (std)
Wave height (mean)
Salinity (mean)
IR albedo (diffuse) (mean)
Q
Bathymetry (mean)
Q_α
PR area growth
Score
Past
Q_α
Q_A
PR area
Distance
−1
−0.5
0
0.5
1

(a)

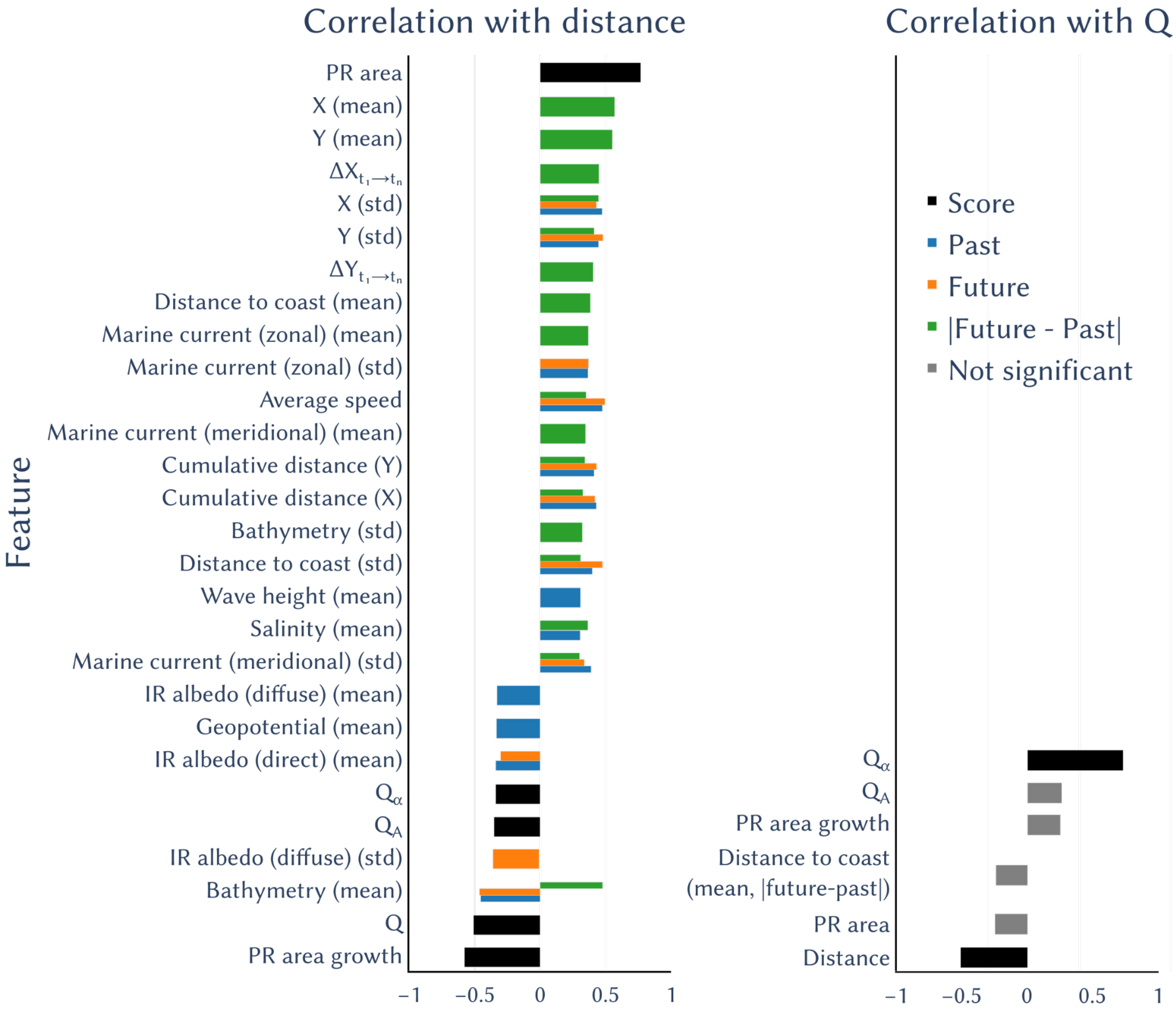

(b)

Figure S7. Spearman correlation of trajectory properties with distance error and PR quality. Significant Spearman correlations corrected for multiple comparisons using the Benjamini-Hochberg method, controlling for a false discovery rate 0.05. between average characteristics for trajectory and the prediction regions, and the obtained point prediction error (left) and prediction region quality (right), for the forecasting **(a)** and imputation **(b)** tasks. Correlations involving point prediction and PR scores are plotted in black, past features in blue, future features in orange and absolute differences between future and past features in green. Correlations that were initially significant but did not pass the multiple comparisons correction are shown in gray. The performance of the point prediction and the PR are interconnected, with a Spearman correlation between the distance error and Q of -0.495 for forecasting and -0.51 for imputation.

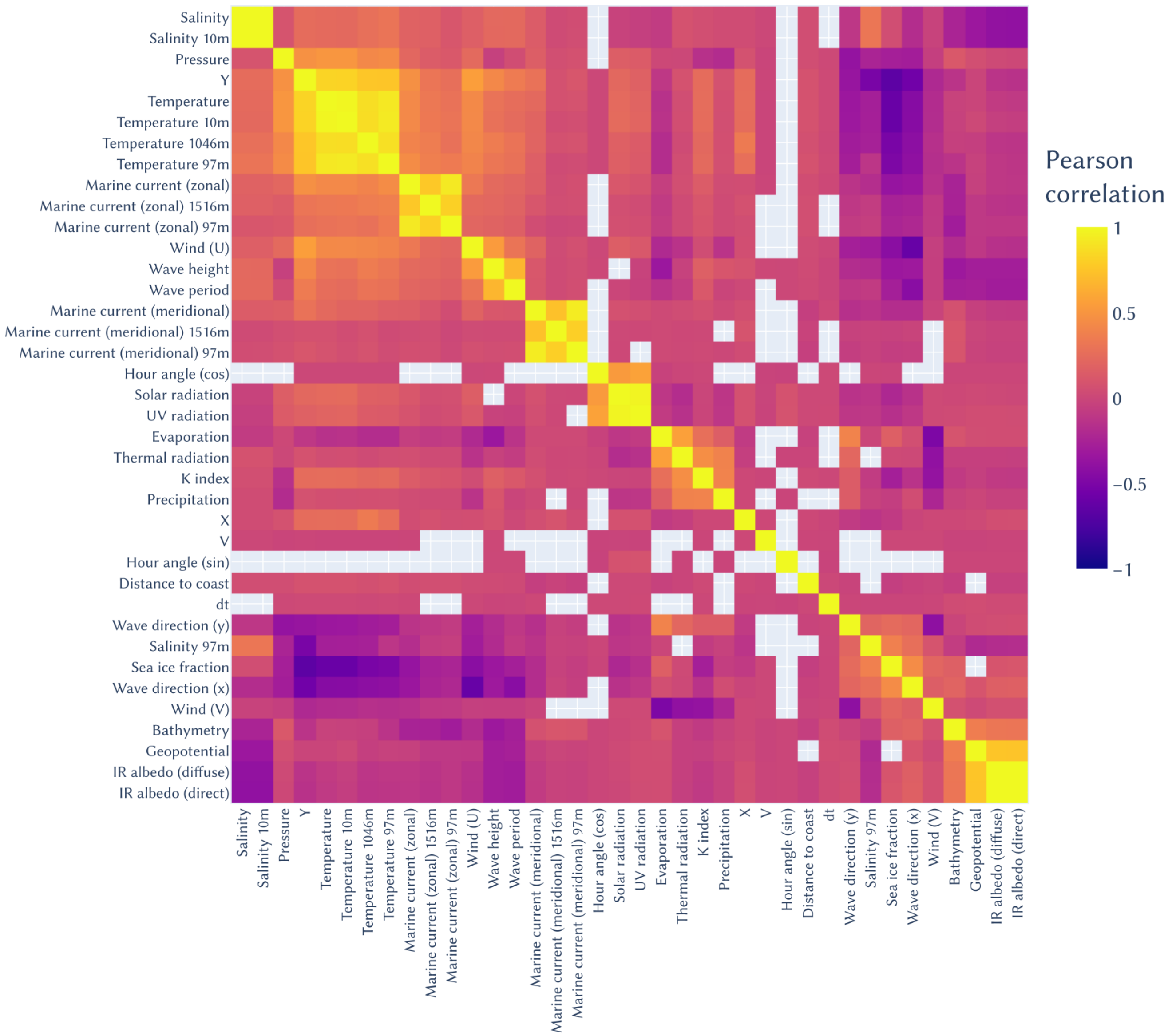

Figure S8. Collinearity plot. Hierarchical clustering of Pearson correlations among environmental features, using the "average" method (UPGMA). Non-significant correlations (p > 0.05) are not colored.

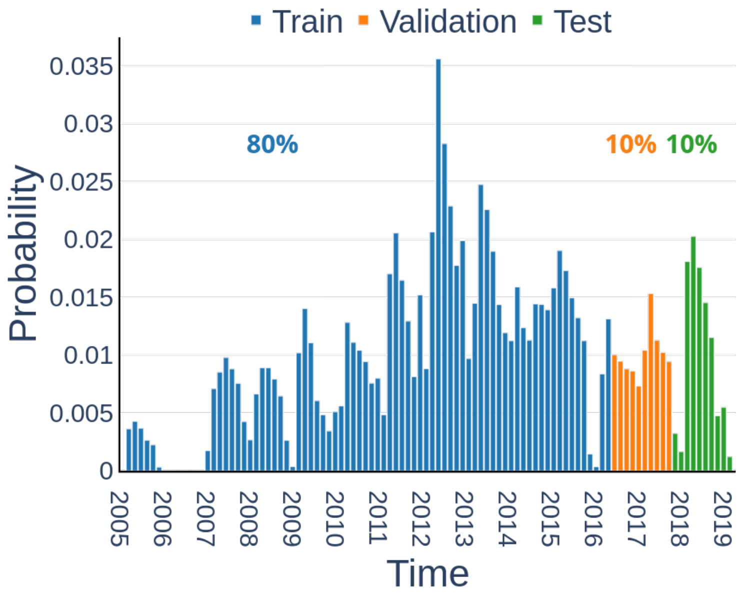

(a)

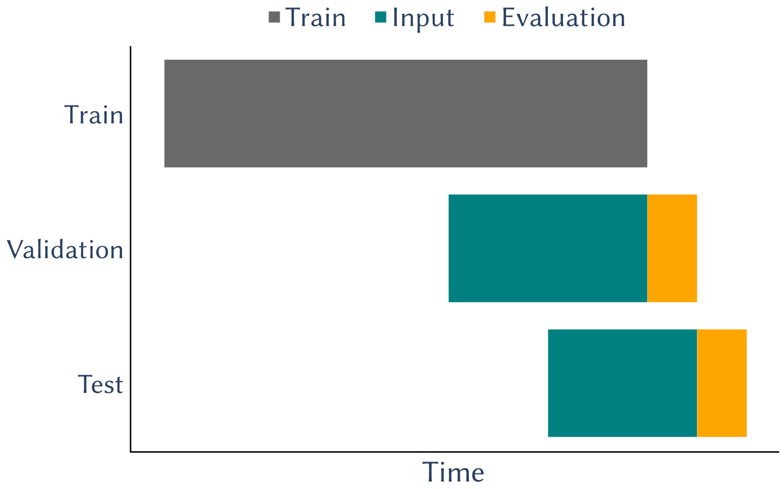

(b)

Figure S9. Chronological train-val-test split. (a) Full dataset. To avoid look-ahead bias, the dataset is divided in chronological order: the first 80% for training, the next 10% for validation, and the final 10% for testing. **(b) Single trajectory.**To avoid look-ahead bias and maximize the training data, the trajectory is divided in chronological order allowing for overlap in the input regions but not in the evaluation region, limiting data leakage.

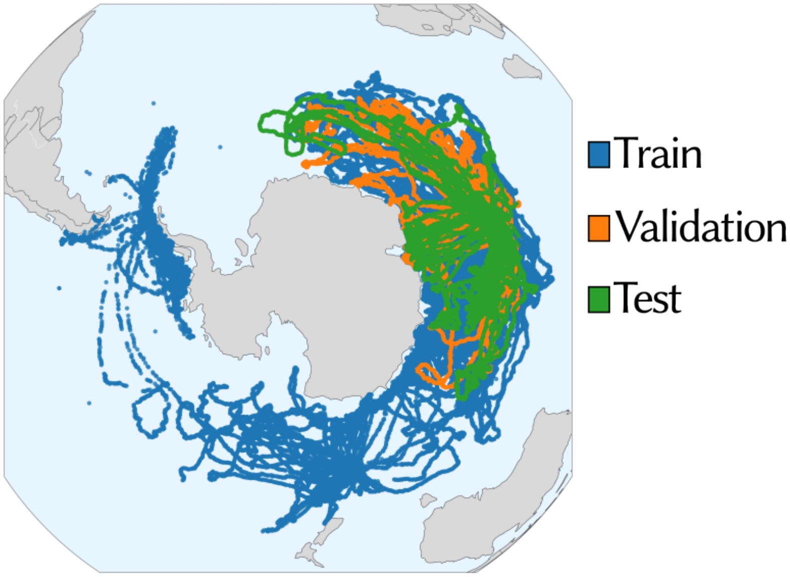

(a)

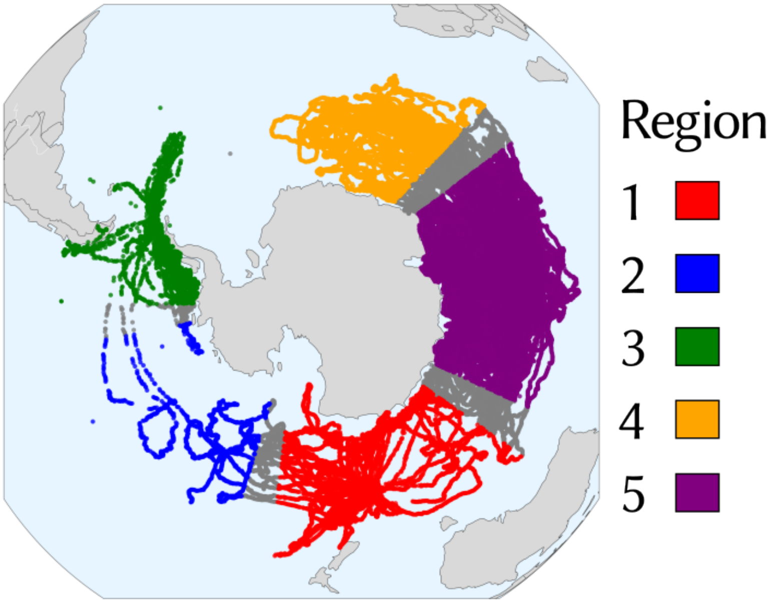

(b)

Figure S10. Geographical visualization of the dataset splits. (a) Chronological. To avoid look-ahead bias, the dataset is divided in chronological order: the first 80% for training, the next 10% for validation, and the final 10% for testing. Geographically, the training, validation and test distribution overlap. **(b) Geographical.** To quantify the geographical bias, data is splitted in five geographical regions. Four of them are used for training and validation, and the remaining one is used to test the performance in an unseen region. Gray areas are boundary zones of width 10 degrees that are not assigned to any cluster.

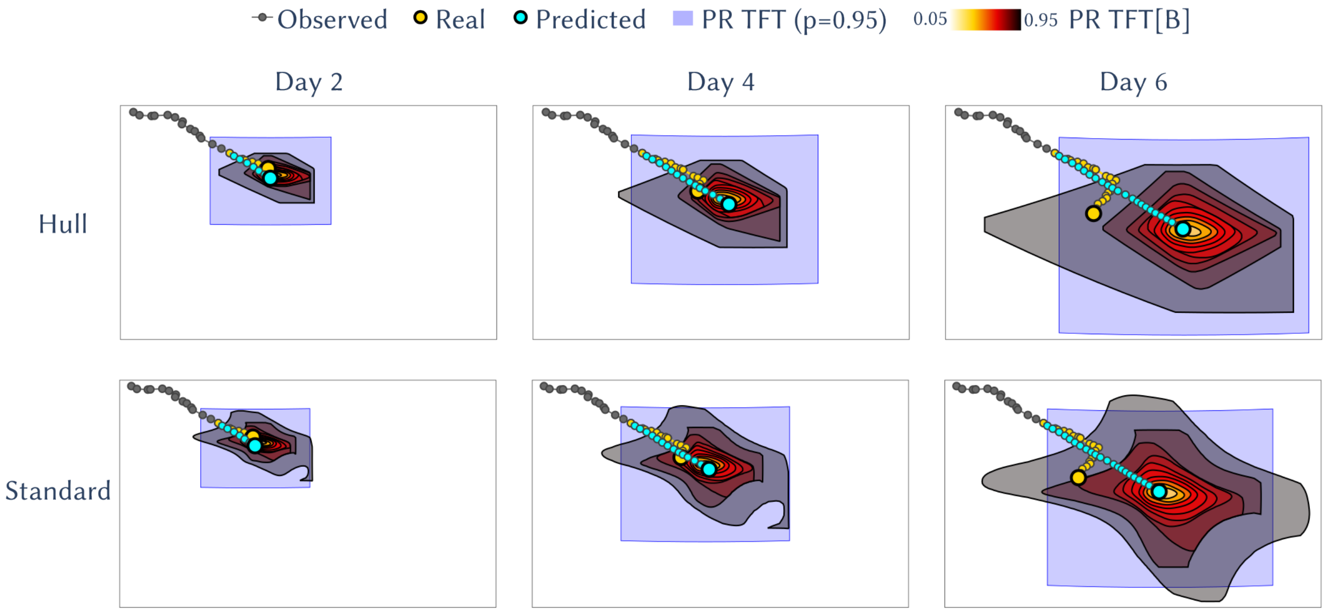

(a)

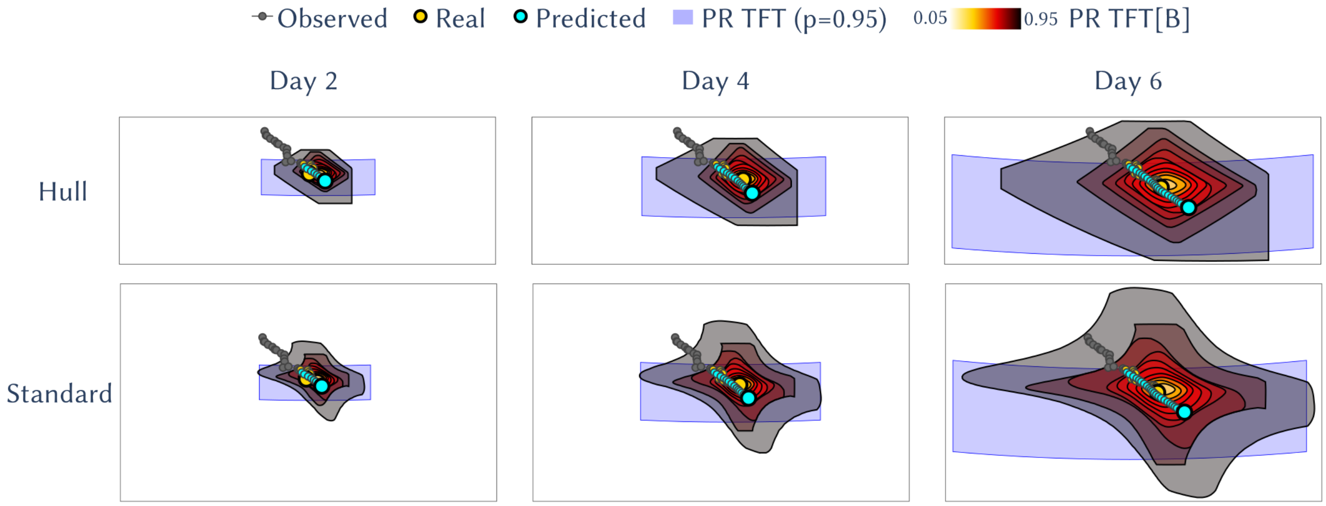

(b)

Figure S11. Example outputs for the forecasting task. Equivalent of Fig. 1 for other trajectories, using the Hull (above) and standard (below) procedures for the PRs estimated from the bivariate distribution.

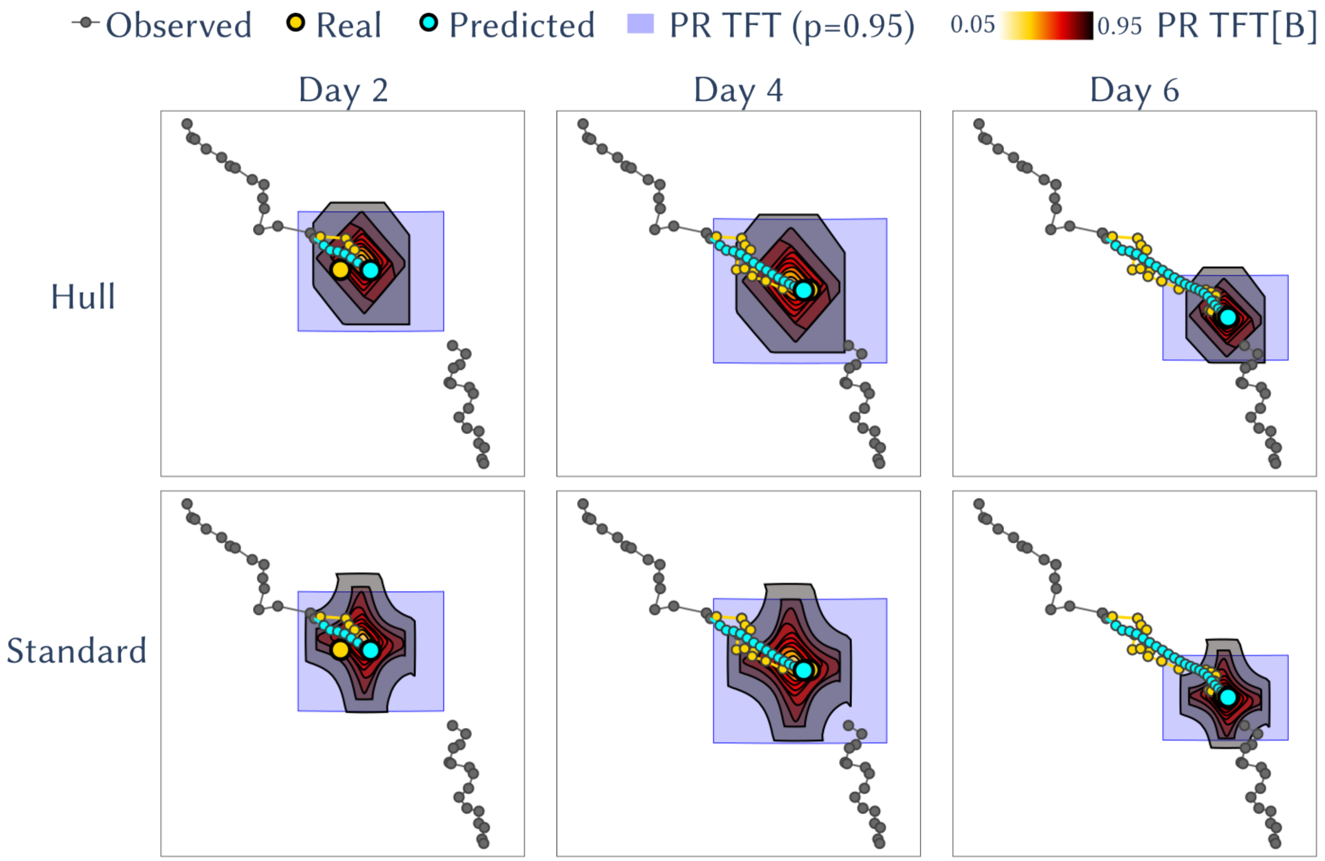

(a)

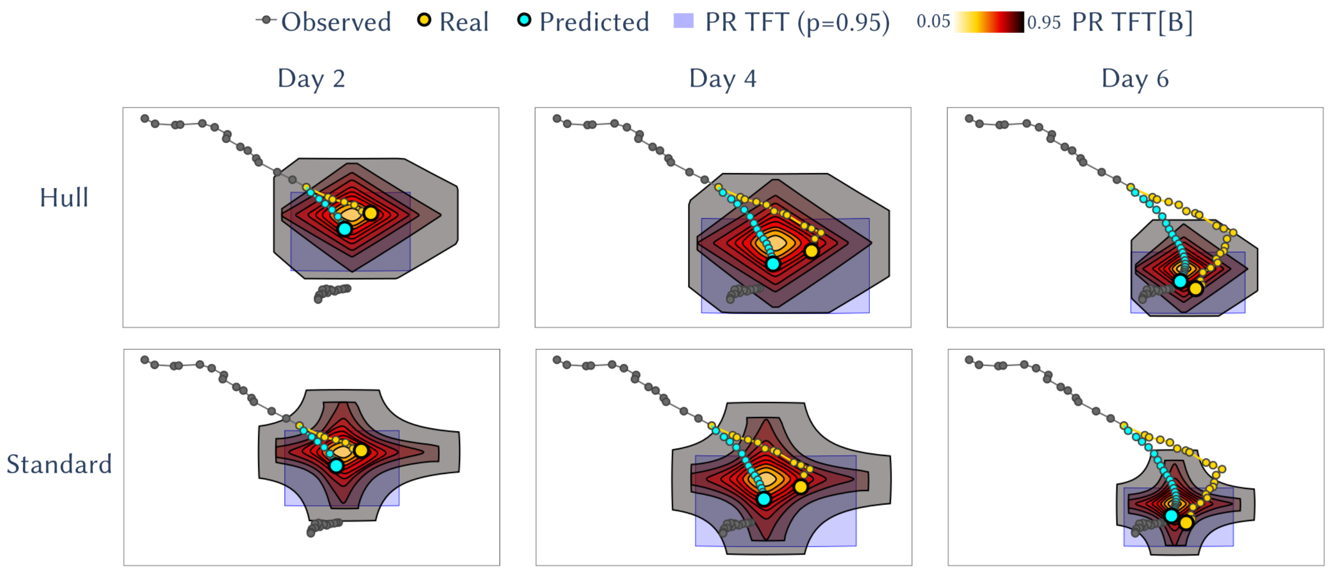

(b)

Figure S12. Example outputs for the imputation task. Equivalent of Fig. 3 for other trajectories, using the Hull (above) and standard (below) procedures for the PRs estimated from the bivariate distribution.

	Forecasting		Imputation	
	Top best	Undisputed best	Top best	Undisputed best
TFT[B]	<u>1.0</u>	0	<u>1.0</u>	0
TFT	<u>1.0</u>	0	<u>1.0</u>	0
TFT[s]	0.07	0	0	0
Naive	0.11	0	<u>1.0</u>	0
RW	0.64	0	0.93	0
CRW	0.07	0	0.11	0
MP	0.96	0	0.43	0

Table S1. Percentage of time steps where each model outperforms in terms of distance error. Percentage of time steps in which the distance error (Fig. S5) of each model is among the top best, i.e. indistinguishable with statistical significance from the best performers, or is the undisputed best with statistical significance.

	$\hat{\alpha}(\alpha = 0.05)$	$\hat{\alpha}(\alpha = 0.1)$	$\hat{\alpha}(\alpha = 0.5)$	Area ($\alpha = 0.05$)	Area ($\alpha = 0.1$)	Area ($\alpha = 0.5$)	Distance
**TFT[B]**	0.05 [0.03, 0.08]	0.11 [0.08, 0.14]	0.49 [0.44, 0.54]	**1.85** [1.60, 2.22]	**1.12** [0.97, 1.35]	0.17 [0.15, 0.20]	66 [60, 73]
**TFT**	0.05 [0.03, 0.08]	0.10 [0.07, 0.13]	0.47 [0.43, 0.51]	3.42 [2.95, 4.44]	2.01 [1.73, 2.62]	0.35 [0.29, 0.47]	67 [61, 75]
**TFT[s]**	0.10 [0.03, 0.21]	0.11 [0.05, 0.23]	0.66 [0.53, 0.78]	53.0 [28.4, 106]	41.9 [22.2, 87.0]	1.07 [0.47, 3.00]	163 [122, 234]
**Naive**	0.07 [0.05, 0.09]	0.11 [0.09, 0.14]	0.49 [0.44, 0.53]	119 [95, 148]	35.8 [28.9, 44.5]	0.69 [0.56, 0.85]	107 [95, 121]
**RW**	0.03 [0.02, 0.05]	0.06 [0.04, 0.10]	0.35 [0.30, 0.40]	8.31 [7.04, 10.3]	5.35 [4.53, 6.62]	0.45 [0.38, 0.55]	96 [82, 110]
**CRW**	0.03 [0.02, 0.03]	0.03 [0.02, 0.04]	0.54 [0.47, 0.61]	135 [94, 208]	105 [72.3, 164]	3.12 [2.07, 5.12]	189 [165, 213]
**MP**	0.03 [0.02, 0.07]	0.04 [0.02, 0.08]	0.45 [0.39, 0.51]	8.60 [7.17, 10.8]	5.76 [4.80, 7.21]	0.21 [0.17, 0.26]	80 [70, 94]

Table S2. Performance summary for the forecasting task. Units: Area is measured in 10^5 km² and distance in km. All metrics are averaged across trajectories and time steps. $\hat{\alpha}$ and α are the empirical and target CER, respectively. Underline denotes the models with indistinguishable top performance, while bold highlights the best model at a 95% confidence level. All models verifying $\hat{\alpha} \not> \alpha$ are considered equal in terms of CER performance.

	$\hat{\alpha}(\alpha = 0.05)$	$\hat{\alpha}(\alpha = 0.1)$	$\hat{\alpha}(\alpha = 0.5)$	**Area** $(\alpha = 0.05)$	**Area** $(\alpha = 0.1)$	**Area** $(\alpha = 0.5)$	**Distance**
**TFT[B]**	0.05<br>[0.04, 0.08	0.11<br>[0.09, 0.14]	0.49<br>[0.46, 0.53]	0.19<br>[0.17, 0.22]	0.11<br>[0.10, 0.13]	0.02<br>[0.02, 0.03]	24<br>[22, 27]
**TFT**	0.06<br>[0.05, 0.09]	0.10<br>0.08, 0.12]	0.46<br>[0.43, 0.49]	0.17<br>[0.15, 0.20]	0.12<br>[0.10, 0.14]	0.03<br>[0.02, 0.03]	25<br>[23, 27]
**TFT[s]**	0.04<br>[0.00, 0.17]	0.03<br>[0.00, 0.15]	0.64<br>[0.44, 0.81]	15.9<br>[9.4, 25.7]	13.1<br>[7.6, 20.6]	0.44<br>[0.25, 0.75]	122<br>[93, 155]
**Naive**	0.05<br>[0.03, 0.08]	0.09<br>[0.07, 0.12]	0.44<br>[0.39, 0.48]	8.09<br>[6.41, 12.2]	2.64<br>[2.00, 4.57]	0.11<br>[0.07, 0.26]	28<br>[25, 30]
**RW**	0.03<br>[0.03, 0.04]	0.09<br>[0.08, 0.10]	0.44<br>[0.42, 0.46]	0.30<br>[0.28, 0.32]	0.17<br>[0.16, 0.18]	0.03<br>[0.02, 0.03]	29<br>[28, 30]
**CRW**	0.04<br>[0.03, 0.04]	0.05<br>[0.04, 0.05]	0.54<br>[0.52, 0.56]	1.82<br>[1.62, 2.09]	1.44<br>[1.28, 1.65]	0.09<br>[0.08, 0.10]	43<br>[41, 45]
**MP**	0.03<br>[0.02, 0.04]	0.03<br>[0.03, 0.04]	0.40<br>[0.38, 0.43]	0.81<br>[0.77, 0.86]	0.60<br>[0.57, 0.64]	0.05<br>[0.04, 0.05]	32<br>[30, 34]

Table S3. Performance summary for the imputation task. Units: Area is measured in 10^5 km² and distance in km. All metrics are averaged across trajectories and time steps. $\hat{\alpha}$ and α are the empirical and target CER, respectively. Underline denotes the models with indistinguishable top performance, while bold highlights the best model at a 95% confidence level. All models verifying $\hat{\alpha} \not> \alpha$ are considered equal in terms of CER performance.

Variable	Original	Binned (Δt = 6h)
Trajectories	434	434
Observations	1660 ([1550, 1778])	538 ([508, 568])
Days	182 ([170, 198])	182 ([170, 198])
Sampling period (h)	3.8 ([3.4, 4.4])	8.8 ([8.3, 9.6])

Table S4. Satellite tracking data for southern elephant seals. Tracking data of southern elephant seals from French Polar Institute programs: *SNO-MEMO*(program 109: PI. H. Weimerskirch) and *CNES-TOSCA*(program 1201: P.I. C. Gilbert and C. Guinet); and Australia's Integrated Marine Observing System (*IMOS*). Intervals between brackets correspond to 95% CIs for the mean. Observations and days are rounded the integer value, and the sampling period is rounded to the first decimal.

Dataset	Temporal resolution	Spatial resolution	Variables
ERA5	hourly	0.25° for atmospheric variables, 0.5° for ocean waves	Temperature, radiation, atmospheric stability, moisture, oceanic and geophysical.
ORAS5	montly	0.25°	Oceanic.
Natural Earth	-	1:10m	Ocean coastline.

Table S5. Datasets containing environmental features

Variable	Unit	Description
Temperature	K	Foundation Sea Surface Temperature (SST) which excludes diurnal variations. Different datasets are used for different time periods: HadISST2 before September 2007 and OSTIA afterwards.
Sea ice fraction	-	Fraction of the grid box covered by sea ice, excluding ice formed on land like glaciers, icebergs, ice-sheets, and ice shelves. The IFS model doesn't include these land-based ice forms. Datasets used are OSI SAF (409a) until August 2007 and OSI SAF oper thereafter.
Net solar radiation	Jm^{-2}	Balance of solar radiation (shortwave radiation) received versus reflected by the Earth's surface, calculated over a 1-hour period. It includes both direct and diffuse radiation.
Net thermal radiation	Jm^{-2}	Difference between downward and upward longwave (thermal) radiation at the Earth's surface. It accounts for radiation emitted by the atmosphere, clouds, and the Earth's surface, calculated over a 1-hour period.
IR albedo for diffuse radiation	-	Albedo here measures the reflectivity of the Earth's surface for diffuse solar radiation in the 0.7 to 4 µm wavelength range, applicable to snow-free land surfaces. It varies seasonally and is modeled differently for water, ice, and snow surfaces in the ECMWF Integrated Forecasting System (IFS).
IR albedo for direct radiation	-	Similar to the diffuse radiation, this albedo measures the Earth's surface reflectivity for direct solar radiation in the 0.7 to 4 µm wavelength range, also applicable to snow-free land surfaces. The IFS adjusts this seasonally for various surfaces.
Downward UV radiation	Jm^{-2}	Ultraviolet (UV) radiation in the 0.20-0.44 µm wavelength range that reaches the Earth's surface. Despite some absorption by the ozone layer, UV radiation can have harmful effects on cells and human health. The UV radiation level is accumulated over 1 hour for reanalysis and 3 hours for ensemble mean and spread. The unit used is joules per square meter (Jm^{-2}), and it can be converted to watts per square meter (Wm^{-2}) for different accumulation periods.

Table S6. Temperature and radiation-related variables. Retrieved from the *ERA5 hourly data on single levels from 1979 to present* dataset.

Variable	Unit	Description
K index	K	Potential for a thunderstorm to develop, calculated from the temperature and dew point temperature in the lower part of the atmosphere. The calculation uses the temperature at 850, 700 and 500 hPa and dewpoint temperature at 850 and 700 hPa. Higher values of K indicate a higher potential for the development of thunderstorms: <20 K No thunderstorm, 20-25 K Isolated thunderstorms, 26-30 K Widely scattered thunderstorms, 31-35 K Scattered thunderstorms, >35 K Numerous thunderstorms.
Pressure	Pa	atmospheric pressure at the Earth's surface, adjusted to mean sea level. Mean sea level pressure maps help identify low and high-pressure systems (cyclones and anticyclones). The contours of mean sea level pressure also indicate wind strength.
Total precipitation	m	Accumulated depth of liquid and frozen water falling to the Earth's surface, representing the depth as if it were spread evenly over a grid box. It includes both large-scale and convective precipitation. Large-scale precipitation arises from cloud formation and dissipation due to atmospheric changes, as predicted by the ECMWF Integrated Forecasting System (IFS) at or above grid box scales. Convective precipitation, on the other hand, results from smaller-scale convection. This parameter excludes fog, dew, and precipitation that evaporates before reaching the surface. The accumulation period for reanalysis data is the hour leading up to the validity date and time.
Evaporation	m	Accumulated amount of water evaporated from the Earth's surface, including a simplified representation of transpiration from vegetation. The accumulation period varies depending on the data extracted: for reanalysis data, it is the hour ending at the validity date and time; for ensemble members, ensemble mean, and ensemble spread, it's over the 3 hours ending at the validity date and time. In the ECMWF IFS convention, negative values indicate evaporation and positive values indicate condensation.

Table S7. Atmospheric Stability and Moisture Variables. Retrieved from the *ERA5 hourly data on single levels from 1979 to present* dataset.

Variable	Unit	Dataset	Description
Mean wave direction	degree true	ERA5	Mean over all frequencies and directions of the two-dimensional wave spectrum, a combination of waves with different heights, lengths and directions.
Mean wave period	s	ERA5	Average time for two consecutive wave crests, on the surface of the ocean/sea, to pass through a fixed point.
Significant height of combined wind waves and swell	m	ERA5	Average height of the highest third of surface ocean/sea waves generated by wind and swell. It is computed as four times the square root of the integral of the two-dimensional wave spectrum over all directions and frequencies. This height represents the vertical distance between the crest and trough of a wave.
Marine current (meridional)	ms^{-1}	ORAS5	Northward (meridional) horizontal surface velocity of a water parcel at a depth of 1 meter, with adjustments made for rotation from the model grid to a latitude and longitude grid.
Marine current (zonal)	ms^{-1}	ORAS5	Eastward (zonal) horizontal surface velocity of a water parcel at a depth of 1 meter, with adjustments made for rotation from the model grid to a latitude and longitude grid.
Salinity	PSU	ORAS5	Salt content of sea water measured at a depth of 1 meter. It is quantified in practical salinity units (PSU).

Table S8. Oceanic variables. Retrieved from the *ERA5 hourly data on single levels from 1979 to present* and *ORAS5 global ocean reanalysis monthly data from 1958 to present* datasets. *Degree true: direction relative to the north pole. Thus, 0 degrees means "coming from the north" and 90 degrees means "coming from the east".

Variable	Unit	Dataset	Description
Geopotential	m^2s^{-2}	ERA5	Gravitational potential energy per unit mass at a specific location on the Earth's surface relative to mean sea level. It indicates the amount of work required to lift a unit mass from mean sea level to that location against the force of gravity. The surface geopotential height (or orography) is derived by dividing the surface geopotential by the Earth's gravitational acceleration, which is approximately 9.80665 m/s^2. It is a time-invariant parameter
Bathymetry	m	Natural Earth	Underwater depth of the ocean.
Distance to coast	km	Natural Earth	Distance to the nearest ocean coastline, including major islands. The coastline data is matched to land and water polygons, primarily derived from World Data Bank 2, with some generalization applied through line simplification in Adobe Illustrator. For the Antarctic coast, the data comes from NASA's Mosaic of Antarctica.

Table S9. Geophysical and topographical variables Retrieved from the *ERA5 hourly data on single levels from 1979 to present* and *Natural Earth 10m-coastline* datasets.

Variable	Description
Month	Category in [0, 11].
Season	Category in {winter, spring, summer, autumn}.
Time index	Integer in [-encoder length, prediction length]. Encoder refers to the observed, past data. In our case, with four observations per day (sampling rate = 6 hours), observed days in [14,28] and predicted days in [1/4,7], the encoder length is within [56, 112] and the prediction length is within [1, 28]. For the validation and test sets, the prediction length is always 28.
$\cos \tilde{t}$	Cosinus component of the rescaled day of the year.
$\sin \tilde{t}$	Sinus component of the rescaled day of the year.

Table S10. Decoder variables. Decoder input variables. Their values are known during the prediction time window. These variables are also part of the encoder input.

Variable	Description
Sex	Category in {M, F, U}. U for unknown sex.
Weight	Weight of the animal.
Length	Length of the animal.
Encoder length	Number of past observed data points.
Future length	Number of future observed data points. Used only in the imputation task.

Table S11. Static variables. Metadata that does not vary with time. The TFT model considers these variables as categoricals.

Parameter	Search space	Search type	Optimal forecasting	Optimal imputation
gradient_clip_val	[0.01, 100.0]	loguniform	7.37 (7.22)	79.95 (22.56)
lstm_layers	[1, 4]	int	1 (1)	2 (1)
hidden_size	[32, 512]	int	250 (260)	317 (787)
hidden_continuous_size	[4, 128]	int	58 (39)	128 (425)
attention_head_size	[1, 32]	int	11 (2)	7 (6)
dropout	[0, 0.3]	uniform	0.1 (0.1)	0.0005 (0.1)
learning_rate	[10^{-4}, 0.1]	loguniform	0.003 (0.003)	0.001 (0.0004)
maximum input days	[1, 84]	int	4 (4)	4 (4)
dq	{0.005, 0.01, 0.05, 0.1}	categorical	(0.05)	(0.01)

Table S12. Hyperparameter search for Temporal Fusion Transformer Hyperparameter search for TFT using the 'optuna' Python package. The first three columns specify the parameter space, while the final two columns are the optimal hyperparameters for the forecasting and imputation tasks. In these columns, the value on the left corresponds to TFT, while the value in parentheses represents TFT[B]. For the learning rate we use the tuner.lr_find method from pytorch lightning's Trainer class, which falls back to optuna if any error is raised. Dropout is only optimized for lstm_layers greater than 1, otherwise it is set to the default value 0.1.

Model	Q_α	Q_A	Q
TFT[B]	0.93 [0.87, 0.98]	0.76 [0.74, 0.78]	**0.71 [0.67, 0.75]**
TFT	0.91 [0.83, 0.97]	0.67 [0.65, 0.69]	0.61 [0.56, 0.65]
TFT[s]	0.21 [0.13, 0.31]	0.69 [0.59, 0.77]	0.13 [0.09, 0.20]
Naive	0.02 [0.02, 0.02]	**0.99 [0.98, 0.99]**	0.02 [0.01, 0.02]
RW	0.70 [0.53, 0.86]	0.45 [0.40, 0.51]	0.33 [0.27, 0.38]
CRW	0.97 [0.95, 0.98]	0.10 [0.08, 0.13]	0.08 [0.06, 0.10]
MP	0.95 [0.85, 0.98]	0.30 [0.25, 0.36]	0.27 [0.24, 0.33]

Table S13. Quality metrics for the forecasting task without increasing the prediction region boundaries. Equivalent of Table 1 without tuning the size of the PR. Sample average and 95% CI between brackets for the point prediction error (first column) and prediction region performance (last three columns) for each model. Underline denotes the models with indistinguishable top performance, while bold highlights the best model at a 95% confidence level. The point prediction error is the shortest distance between the real point and the predicted one, averaged across trajectories and time steps. Prediction region performance is assessed using the quality metrics: coverage error rate (Q_α), area (Q_A), and overall quality (Q), averaged across trajectories, time steps, and target coverage error rates $\alpha_F \in \{0.05, 0.1, 0.5\}$.

	$\hat{\alpha}(\alpha = \mathbf{0.05})$	$\hat{\alpha}(\alpha = \mathbf{0.1})$	$\hat{\alpha}(\alpha = \mathbf{0.5})$	**Area** $(\alpha = \mathbf{0.05})$	**Area** $(\alpha = \mathbf{0.1})$	**Area** $(\alpha = \mathbf{0.5})$	**Distance**
**TFT[B]**	0.08 [0.06, 0.11]	<u>0.10</u> [0.07, 0.13]	<u>0.50</u> [0.46, 0.55]	1.37 [1.18, 1.64]	1.20 [1.04, 1.44]	0.16 [0.14, 0.19]	<u>66</u> [60, 73]
**TFT**	<u>0.06</u> [0.04, 0.09]	<u>0.12</u> [0.10, 0.15]	<u>0.54</u> [0.49, 0.58]	2.81 [2.42, 3.64]	1.68 [1.44, 2.19]	0.26 [0.22, 0.36]	<u>67</u> [60, 74]
**TFT[s]**	0.50 [0.34, 0.64]	0.61 [0.47, 0.74]	0.89 [0.82, 0.94]	1.63 [0.88, 3.26]	1.26 [0.67, 2.60]	0.43 [0.19, 1.19]	162 [121, 233]
**Naive**	0.95 [0.94, 0.96]	0.96 [0.95, 0.97]	0.98 [0.98, 0.99]	**0.00** [0.00, 0.00]	**0.00** [0.00, 0.00]	**0.00** [0.00, 0.00]	107 [94, 121]
**RW**	0.23 [0.18, 0.28]	0.25 [0.20, 0.31]	<u>0.39</u> [0.34, 0.43]	4.44 [3.99, 5.18]	3.41 [3.07, 3.98]	1.03 [0.92, 1.20]	95 [81, 110]
**CRW**	<u>0.03</u> [0.02, 0.03]	<u>0.03</u> [0.02, 0.04]	<u>0.10</u> [0.08, 0.13]	337 [255, 485]	259 [196, 373]	78.1 [59.0, 112]	188 [165, 212]
**MP**	<u>0.06</u> [0.04, 0.09]	<u>0.09</u> [0.06, 0.12]	<u>0.19</u> [0.15, 0.23]	13.6 [12.0, 15.6]	10.4 [9.23, 12.0]	3.14 [2.78, 3.62]	<u>80</u> [70,94]

Table S14. Performance summary for the forecasting task without increasing the prediction region boundaries. Units: Area is measured in 10^5 km² and distance in km. All metrics are averaged across trajectories and time steps. $\hat{\alpha}$ and α are the empirical and target CER, respectively. Underline denotes the models with indistinguishable top performance, while bold highlights the best model at a 95% confidence level. All models verifying $\hat{\alpha} \not> \alpha$ are considered equal in terms of CER performance.

Model	Q_α	Q_A	Q
TFT[B]	0.90 [0.85, 0.95]	0.89 [0.88, 0.91]	0.81 [0.76, 0.85]
TFT	0.90 [0.84, 0.95]	0.90 [0.89, 0.92]	0.82 [0.77, 0.86]
TFT[s]	0.30 [0.15, 0.49]	0.59 [0.47, 0.70]	0.18 [0.09, 0.31]
Naive	0.03 [0.02, 0.04]	**0.99 [0.98, 1.00]**	0.03 [0.02, 0.04]
RW	0.97 [0.97, 0.98]	0.42 [0.40, 0.43]	0.39 [0.37, 0.41]
CRW	0.96 [0.95, 0.97]	0.26 [0.24, 0.28]	0.23 [0.21, 0.25]
MP	0.97 [0.96, 0.97]	0.29 [0.28, 0.31]	0.27 [0.25, 0.28]

Table S15. Quality metrics for the imputation task without increasing the prediction region boundaries. Equivalent of Table 2 without tuning the size of the PR. Sample average and 95% CI between brackets for the point prediction error (first column) and prediction region performance (last three columns) for each model. Underline denotes the models with indistinguishable top performance, while bold highlights the best model at a 95% confidence level. The point prediction error is the shortest distance between the real point and the predicted one, averaged across trajectories and time steps. Prediction region performance is assessed using the quality metrics: coverage error rate (Q_α), area (Q_A), and overall quality (Q), averaged across trajectories, time steps, and target coverage error rates $\alpha_F \in \{0.05, 0.1, 0.5\}$.

	$\hat{\alpha}(\alpha = \mathbf{0.05})$	$\hat{\alpha}(\alpha = \mathbf{0.1})$	$\hat{\alpha}(\alpha = \mathbf{0.5})$	**Area** ($\alpha = \mathbf{0.05}$)	**Area** ($\alpha = \mathbf{0.1}$)	**Area** ($\alpha = \mathbf{0.5}$)	**Distance**
**TFT[B]**	0.09 [0.07, 0.11]	<u>0.10</u> [0.08, 0.13]	<u>0.52</u> [0.48, 0.55]	0.13 [0.12, 0.15]	0.12 [0.10, 0.13]	0.02 [0.02, 0.02]	<u>24</u> [22, 27]
**TFT**	0.08 [0.06, 0.10]	0.13 [0.11, 0.16]	<u>0.51</u> [0.48, 0.55]	0.15 [0.13, 0.17]	0.10 [0.09, 0.12]	0.02 [0.02, 0.03]	<u>25</u> [23, 27]
**TFT[s]**	041 [0.24, 0.60]	0.49 [0.30, 0.69]	0.83 [0.67, 0.93]	1.17 [0.68, 1.91]	0.91 [0.53, 1.43]	0.15 [0.08, 0.26]	122 [93, 155]
**Naive**	0.92 [0.90, 0.94]	0.94 [0.92, 0.95]	0.98 [0.97, 0.99]	**0.00** [0.01, 0.01]	**0.00** [0.00, 0.01]	**0.00** [0.00, 0.00]	<u>28</u> [25, 30]
**RW**	<u>0.02</u> [0.02, 0.03]	<u>0.04</u> [0.04, 0.05]	<u>0.21</u> [0.20, 0.23]	1.15 [1.09, 1.22]	0.89 [0.84, 0.94]	0.27 [0.25, 0.28]	29 [28, 30]
**CRW**	<u>0.04</u> [0.03, 0.04]	<u>0.05</u> [0.04, 0.05]	<u>0.16</u> [0.14, 0.17]	6.26 [5.68, 6.99]	4.81 [4.37, 5.37]	1.45 [1.31, 1.62]	43 [41, 45]
**MP**	<u>0.03</u> [0.02, 0.04]	<u>0.03</u> [0.03, 0.04]	<u>0.11</u> [0.10, 0.13]	2.86 [2.69, 3.05]	2.20 [2.07, 2.35]	0.66 [0.62, 0.71]	32 [30, 34]

Table S16. Performance metrics for the imputation task without increasing the prediction region boundaries. Units: Area is measured in 10^5 km^2 and distance in km. All metrics are averaged across trajectories and time steps. $\hat{\alpha}$ and α are the empirical and target CER, respectively. Underline denotes the models with indistinguishable top performance, while bold highlights the best model at a 95% confidence level. All models verifying $\hat{\alpha} \not> \alpha$ are considered equal in terms of CER performance.
